# Nursing team perceptions in caring for sex workers in primary care

**DOI:** 10.1590/1980-220X-REEUSP-2024-0331en

**Published:** 2025-04-11

**Authors:** Helen Prates Rodrigues, Giulia Delfini, Aldair Weber, Ana Paula Rigon Francischetti Garcia, Vanessa Pellegrino Toledo

**Affiliations:** 1 Universidade Estadual de Campinas, Faculdade de Enfermagem, Campinas, São Paulo, SP, Brazil.

**Keywords:** Nursing, Team, Nursing Care, Sex Workers, Access to Primary Care

## Abstract

**Objective:**

To understand nursing team perceptions of a Basic Health Unit in a city in the countryside of São Paulo regarding the care provided to sex workers.

**Method:**

This is qualitative research based on Alfred Schutz’s framework, with semi-structured interviews conducted with 12 nursing professionals.

**Results:**

The category “Nursing team perceptions and their lifeworld in caring for sex workers” revealed the reasons “why”, highlighting the challenges faced in nursing care in vulnerable contexts. The reasons “for” are present in the category “The intentionality of the care provided”, which highlights the search for problem-solving care and autonomy promotion in sex workers’ lifeworld.

**Conclusion:**

Nursing team perceptions of the care provided recognize these professionals’ needs and, through bonding and support, seek strategies that promote access to healthcare services.

## INTRODUCTION

Sex workers comprise a wide diversity of individuals, carrying out their work in different locations and sociocultural contexts^(1)^. Globally, they face severe health and human rights inequalities, including exposure to violence, sexually transmitted infections (STIs), and unmet sexual and reproductive healthcare needs due to weaknesses in primary care^(1)^.

In Brazil, about 20 years ago, sex workers were officially recognized as an occupation by the Brazilian Classification of Occupations of the Ministry of Labor and Employment^(2)^. Although the activity is legally approved, its organization and operation depend entirely on them, who remain exposed to various vulnerabilities^(3)^.

This scenario occurs due to sex workers’ experiences within the health-disease process, making them vulnerable people. In everyday life, there are aggravating factors of this dynamic due to failures of actions at the social, individual and programmatic levels, such as the neglect of the State, the need for exposure to survive, precarious housing and work conditions and social stigma^(4)^. Therefore, these professionals’ health is constantly at risk, exposing themselves to STIs through sexual relations without the use of condoms, alcohol and drug abuse, psychological stress caused by physical, verbal and sexual violence, in addition to a harmful work environment^(4)^.

When seeking healthcare services, these professionals face situations of institutional violence, encountering barriers in accessing care or being limited to addressing specific issues, such as the use of condoms and contraceptives. The lack of health policies aimed at comprehensive care for this population, together with prejudices and social marginalization, contributes to fragmented care and increased inequity in access^(5)^.

Considering this population’s health needs and vulnerabilities, the right of access to Brazilian Health System (In Portuguese, *Sistema Único de Saúde* - SUS) services is guaranteed to anyone, with Primary Healthcare (PHC) being the main preferential entry point into the country’s Healthcare Network, providing an ideal scenario for continuous, longitudinal and people-centered care^(6)^. User access to the health system depends on organizational and geographic factors, as the conditions of people and services influence their use and continuity of care^(7)^.

In this context, nursing presents itself as an important leading actor in PHC organization and functioning, as it seeks to guarantee initial access, health follow-up, comprehensiveness in actions, in addition to coordination, family and community guidance, and cultural aspects^(8)^. These elements promote a bond between professionals and the population, aiming to offer welcoming, effective and non-discriminatory care, based on principles of promotion, prevention, treatment and rehabilitation of people^(8)^. Thus, nursing stands out as a precursor of comprehensive care for sex workers, from identifying the territory, meeting their needs and ensuring continuity of healthcare.

Therefore, this study is justified by the need to promote comprehensive care for vulnerable populations and ensure universal access to healthcare services^(4,5)^. Although sex workers are officially recognized as an occupation in Brazil, they still face multiple vulnerabilities, such as exposure to STIs, violence and precarious working conditions^(4,5,6)^. The absence of health policies aimed at comprehensive care, combined with social stigma and prejudice in care, contributes to fragmented and insufficient care^(1,7)^. In this context, the role of nursing in PHC is important to build continuous, humanized care focused on these professionals’ sociocultural needs^(8)^. However, there are still gaps in knowledge about care for this population, especially regarding the lack of specific policies, the need to identify and reduce barriers to access to healthcare services, and the importance of combating social marginalization, stigma and prejudice present in the care provided to sex workers^(1,4–8)^.

In view of this problem, researchers are concerned about the importance of understanding nursing team experiences that works in a territory marked by a high confluence of sex workers. This concern is further justified by PHC consolidation as the preferred gateway to SUS, which requires health teams to exercise their leading role in providing comprehensive, inclusive, equitable care without discrimination in people’s access to the care network. This study, therefore, seeks to understand nursing team perceptions about the care provided to sex workers in the territory of a Basic Health Unit (BHU) in a city in the countryside of São Paulo.

## METHOD

### Study Design

This is a qualitative study, based on the theoretical-methodological framework of Alfred Schutz’s phenomenology, which aims to reveal the phenomenon through social action, understood as an intentional act composed of motivations projected by individuals in their lifeworld, permeated by face-to-face intersubjective relationships and their biographical situation, shaping the knowledge base^(9)^. The COnsolidated criteria for REporting Qualitative research (COREQ) recommendations were followed^(10)^.

### Site

The study was conducted at a BHU in a city in the countryside of São Paulo, a service that is part of the public health network and is located in a region created by the government in 1967, based on an urban sanitation process that consisted of removing sex workers and brothels from the city center, relocating them to a remote area. This action aimed to spatially and geographically separate what was considered “dirty or inappropriate” for family life^(11)^.

In the current urban configuration, the area covered by BHU concentrates prostitution establishments, mainly in the Jardim Itatinga neighborhood, which defines the landscape of the place^(11)^. The neighborhood is located in the southwest region of the municipality, about ten kilometers from the center, in an economically devalued area that has the largest number of low-income residents^(11)^. It is currently considered the largest open-air prostitution zone in Latin America, with a circulation of approximately 2,000 sex workers per month, which allows the operation of numerous brothels of different sizes^(12)^.

Therefore, the area covered by BHU has characteristics of exclusion, marginalization and stigma of the population and sex workers in relation to the rest of the city, since it was chosen because, at the time, it was an isolated place, poorly urbanized and difficult to access, marking this public with social and moral vulnerability^(12)^. Currently, BHU is located on the outskirts of the area of the territory that concentrates prostitution establishments, and has Family Health Strategy teams to offer services with professionals from the multidisciplinary team, assistance services, diagnostic support exams, health surveillance and dispensing of medications^(13)^.

It is worth noting that the researchers involved in this study develop their research, teaching and nursing care practices in various health facilities in the municipality, from PHC to specialized services. Thus, indirect and direct care for sex workers occurs daily in these different spaces, bringing proximity to the topic addressed.

### Population

All nursing professionals from a BHU participated in this study, including nurses, nursing technicians and assistants. Access to participants began with the presence of the main researcher at a team meeting at BHU, where the study was presented and the invitation to participate was made. At that time, one professional agreed to participate, who characterized the first participant in the study and who subsequently indicated the next professional who, in turn, also agreed to participate and indicated the next one. This process was repeated until all members of the nursing team had participated in the research.

### Selection Criteria

Members of the nursing team who had experience caring for sex workers in the studied area were included. Professionals on leave or vacation were excluded.

### Data Collection

Data collection was carried out in April and May 2021, using phenomenological interview, which allows the subject who experiences the phenomenon to share with the interviewer the meaning of the action developed in their world of intersubjective relationships^(14)^. The triggering questions were “Have you ever cared for a sex worker in this service?”, “Tell me how it was” and “What was your intention in providing this care?”, and, based on these, other questions were developed that allowed the interviewee to delve deeper into the topic.

The interviews took place in a private location on BHU premises, and were conducted by the main researcher. They were recorded in digital audio, lasting an average of 15 minutes, with no refusal by participants. Data collection was concluded when the researcher understood that the phenomenon had been revealed and her concerns had been resolved, and no new elements had been identified in the constitution of the categories that represent the type of experience lived^(15)^. The research team was inserted into BHU during data collection and, later, when providing feedback to the service regarding the study developed.

### Data Analysis and Processing

Based on the social phenomenology theoretical framework^(9)^, the data were analyzed and categorized by the researchers who contributed to the study, without the use of software. All interviews conducted were analyzed. The stages indicated in studies based on Alfred Schutz’s concepts^(9)^ were observed: full transcription of participants’ statements; careful reading and rereading of interviews to understand the meaning of nursing team professional experiences; verification of excerpts that include common meanings of participants’ experiences; and organization of speeches into thematic categories that demonstrate the “reasons why” and “reasons for” of interviewees’ experiences^(9)^. Social phenomenology and scientific literature on the topic addressed were used to carry out a comprehensive analysis and discussion of results presented in the categories.

### Ethical Aspects

The research met all the Resolution 466/12 criteria, which regulates research involving human beings, and was approved by the *Universidade Estadual de Campinas* Research Ethics Committee, under Opinion 4,659,425. The research participants signed the Informed Consent Form and the Voice Recording Authorization Form before the interviews began. To guarantee participant anonymity, statements were identified by the letter “I” to represent the word “interviewee”, followed by the number corresponding to the chronological order in which the interviews occurred.

## RESULTS

The study was composed of 12 participants, three nurses and nine nursing technicians, with an average of two years of experience in the unit for nurses and seven years for nursing technicians. The interviews allowed the construction of the “we” relationship, organizing the experiences of the other into two categories: the first highlighted the “reasons why” of participants’ lifeworld, named “Nursing team perceptions of the territory and the healthcare service”; and the reasons for participants’ actions are described in the category “The intentionality of the care provided”.

### Nursing Team Perceptions of the Territory and the Healthcare Service

During the interviews, territory characteristics were reported, which corresponds to an area of prostitution and trafficking, resulting in a distancing of professionals in approach and involvement. The territory determines health problems resulting from the use and sale of psychoactive substances and alcohol. There is also recognition that the population of this territory is marked by vulnerability and difficulty in maintaining bonds.

*It’s not just prostitution that we have here, there’s also drug trafficking. So, there are things you can’t get involved in, because then you run the risk of your life... we’re in a territory where we have to be very careful about what we say, what we do, how we approach people.* (I3)

*And we know that sometimes people arrive without a specific problem, or they don’t use drugs, they don’t use drugs or alcohol, and in order to get on with their lives they get involved. They get involved to a level that leads to drug trafficking, which goes much further than it seems.* (I10)

*I think the routine is the same as in any health center, the only difference is that our population is more exposed and more vulnerable.* (I12)

The nursing team recognized that the work with sex workers was greater when BHU was located in the prostitution area. The proximity of the service to the population favored greater daily care, but now there is no preventive healthcare, and the search for care is done when there is a complaint.

*In the past, when we were in the* [neighborhood], *we had a much greater presence, because they came, they had a demand, we were in prostitution, we were in the red-light district, and today we are not.* (I4)

*Access to daily care used to be greater. Not anymore, now they only come to us when they really have a complaint. There is no preventive care, so I think our leaving the territory made a difference. Although we are still here, for them, physically, this has changed.* (I7)

Concerning service characteristics, it was observed that the service is provided without the need to present an identification document, which is justified by the importance of caring for a person’s health. The service structures care based on spontaneous demand and reception, seeking to meet demands as much as possible to favor the connection and outpatient return.

*They come to be seen without their ID... there are several issues that, if we don’t have a quick wit, trying to work even without their ID, because I’m taking care of a person, then... I know, if I see them, I know who they are, but they might have lied about their name too. So, we’re very careful with this issue of care too. Even if they don’t have their ID, we’ll take care of them, we’ll provide healthcare.* (I3)

*These women do not usually come for scheduled care; they always come spontaneously to welcoming. In general, this is a population that does not need to be scheduled. Most of professionals are from other cities, so they will follow up with us. So, we try to meet as much of the demand that they bring on the day as possible, to try to connect them and bring them to the outpatient clinic.* (I1)

The nursing team points out that sex workers have health problems due to exposure to STIs, mental health problems, a history of abuse and use of psychoactive substances. They also mention that sex workers do not seek out the service because they believe they will be mistreated, and when they do arrive, they abandon treatment or do not wait for the outcome of the care provided.

*They are fragile in terms of health, right, due to exposure to STIs, fragile in terms of mental health, and they are usually people who have been abused. There are many stories of abuse in this environment, because, as I said, maintaining this nightlife is very complicated. So, they find themselves quite lost, and the path is to return to drugs or alcohol.* (I10)

*Many of them don’t even come because they think they’ll be mistreated... they create that protective shell themselves. Many of them end up abandoning treatment. We go after them.* (I11)

*Because they are in a hurry, sometimes, while we are running the rapid test, when we call them to give the result, they have already left.* (I1)

Regarding cultural aspects, the nursing team identified that sex workers are marginalized and judged by their families. They also identify a wealth of prejudices that come from their families. They point out that healthcare professionals are subject to stigmas that result in their refusal to serve certain groups.

*They are very marginalized, right, they are judged a lot, even by their own family.* (I10)

*Regardless of whether we are sex workers or not, we come with a lot of baggage from our family; sometimes, there is prejudice.* (I3)

*We have this stigma of separating people based on what they look like, what they are, because I have worked in several units and there is a lot of this thing like “Oh, I don’t treat mental health patients, I don’t treat homosexuals”. Because it’s not just nursing assistants and nursing technicians who need training, because I have seen professionals with higher education, doctors, making this separation.* (I6)

As for work characteristics of sex workers identified by the nursing team, these include going to work accompanied by a “henchman”, limiting their time at the location, and having their identity withheld by the house where they work. They also report working conditions characterized by imprisonment and payment of fines during their absence.

*Normally, they come with the house’s henchman. So, they have a certain amount of time that they can stay here to be attended to. So, if I take too long, if I’m in a hurry, they don’t wait, they leave. There are some girls whose IDs are kept at the house.* (I3)

*They are subjected to a wide range of working conditions; sometimes they are held in prison. Also, because during the time they are away, they have to pay a fine for not being at home or someone comes to monitor them.* (I1)

In relation to sex workers’ demands, it was observed that the complaints are related to gynecological problems that may prevent continuity of work, infectious conditions, search for medication, violence, accidents with condoms, search for rapid tests and unwanted pregnancies.

*They come to us with questions related to gynecological problems or problems of violence or accidents with condoms.* (I12)

*The prostitutes arrived to us very injured; sometimes they were thrown out of cars, you know... they arrived all skinned and we took care of them.* (I8)

*This is the entrance, because the exposure was, I will take the medication, but the main door is the rapid test.* (I10)

*They always come. Sometimes, they come with a pregnancy that they don’t want.* (I2)

*Sometimes, after two days, it smells bad, and you have to start taking antibiotics because you’re already in pain, and there’s already an infectious process there.* [...] *and another thing is that, during the menstrual period, they use cotton inside the vaginal cavity to be able to have sex. So, it’s common for a girl who couldn’t remove the cotton to come to us and ask us to remove it*. (I3)

The nursing team acknowledges that it does its best work, without discrimination, to welcome, listen, and assess personal and family health history, resolving demands and complaints from sex workers, promoting health and providing guidance regarding risks. The team also conducts an approach to understand patients’ history, the reason for seeking the unit and whether they need any psychological help.

*My job is to welcome, listen and try to resolve issues together with them, providing a solution for the demands they bring to me.* [...] *we have to welcome, listen and try to promote health for them. Provide guidance regarding the risks, and that’s it.* (I2)

*Within what they come to us for, we respond, we try to respond in the best way possible, without any discrimination, right?* (I9)

*You see the patient, health issues related to family history, what they have already had, what type of diseases, you ask about vaccinations, you do everything, and then you go to the part of patients’ main complaint.* (I12)

*First, we approach them, try to understand a little about their story, why they are looking for the service.* [...] *then, try to understand what it is like for them. If everything is okay, if they need some psychological help.* (I3)

Professionals perform procedures according to the complaint brought by patients, provide various guidelines and actively seek to continue and complete treatment.

*We insert a speculum, remove the cotton, provide guidance on the risk, and provide guidance on the menstrual cup. Thus, there are a variety of gynecological guidelines*. (I1)

*Problems with the tests we do for STDs, we take care of STDs, we provide instruction.* [...] *as I said, we have to follow up, because most of them don’t come back. So, we who are following up, we have to stay on top of them so they can finish their treatment.* (I11)

### The Intentionality of the Care Provided

Regarding the expectations of the care provided, the nursing team hopes to promote health for sex workers, reduce the risk of disease transmission, welcome them, ensure they return for care, and have success and quality of life. They also hope that care will be effective and make a difference in that person’s life. Finally, they aim to improve care and protection, reduce exposure, and ensure that they understand the need and right to care for their body.

*I hope it promotes good, promotes health for them.* (I2)

*Reduce the risk of disease transmission... they also feel welcomed* [...] *and return, right, ask for feedback, they have somewhere to look for what they need.* (I5)

*Success! May they be able to have a good quality of life*. (I10)

*I hope it is effective and I hope it makes a difference in that person’s life.* (I1)

*We hope that they can take better care of themselves, protect themselves better, and not expose themselves so much.* [...] *but that’s it, she has to try to understand that she has to take care of her body. Even though she’s a professional, she has the right to take care of her body.* (I12)

## DISCUSSION

The nursing professionals participating in the study reported their experiences in caring for sex workers in the area covered by BHU, with the presence of drug trafficking and prostitution in the area, which influences the nursing team’s attitude when caring for this population. Every action brings with it an expectation of a response to this, and in this scenario, it is possible to observe the territory as the subjective meeting space of the lifeworld of professionals and the people who live in it, such as sex workers^(9)^.

The territory is dynamic, and from the relationships created, it can be a product or producer of actions. Thus, when reading the reality presented to individuals, they are invited to present the way in which they place themselves in the territory, called natural attitude^(9)^. Through this, it is possible to approach care in places that may cause tension and fear to healthcare professionals, due to the presence of violence^(16)^.

In PHC, territory is considered to be a delimited geographical space, with an assigned population, dynamic and constantly changing, subject to multiple variables, such as social vulnerabilities^(16)^. In this space, the health-disease process occurs, resulting from the social determinants of health, characterized as factors related to an individual’s living and working conditions, which can influence the emergence of health problems^(16,17)^. Thus, the territory of BHU in this study is presented as the result of a historical dynamic in constant movement that requires its recognition to carry out healthcare, as its origin dates back to social segregation and sanitation strategies of large urban centers.

However, healthcare professionals at BHU reported difficulties in working in this scenario, such as barriers to accessing the population, maintaining a presence in the territory and maintaining ties. The territory covered by PHC may encounter other distinct divisions of people’s organizations, such as drug trafficking, but it is important to consider that the population that lives there has health and care needs, which requires the recognition and planning of actions to be developed^(16)^.

Participants mentioned that BHU was once located between brothels, which represented a strategy for access and proximity of the population to healthcare services. However, in the current organization, care is provided only through complaints, as it requires sex workers to travel to the healthcare service. It is recommended that the location of BHU should consider geographic aspects to facilitate access for the population, which is often not possible, and the units end up located in spaces that are difficult to access^(18)^.

Therefore, it is up to nursing professionals to collaborate in the construction of strategies that reduce barriers to access for vulnerable populations, starting with the recognition of the territory covered by the healthcare service and going on to the identification of the people who live there. Study participants mention welcoming actions based on spontaneous demand and not imposing the presentation of identification documents as ways of welcoming and guaranteeing access to sex workers in BHU. It is worth noting that ensuring these strategies is essential to guarantee people’s right to SUS, resulting from investments in multidisciplinary teams’ work process, whose active presence of professionals is indispensable, since, together, they seek to establish a relationship of trust with users, promoting the construction of solutions and forms of care^(19)^.

Furthermore, participants pointed out that sex workers’ health needs are permeated by exposure to STIs, substance abuse, violence and mental health issues. Studies indicate that sex workers’ mental health issues are often mediated by the place where they work and their vulnerability to violence, substance abuse and stigma^(1,20)^. STIs are an aggravating factor for the mental health of this population, as it is a complex and multifactorial phenomenon, the main measures of which are aimed at prevention, early detection and timely treatment, enhanced by access to healthcare services^(21,22)^. All of these events associated with and experienced by sex workers constitute their life biography, an important aspect for the nursing team when thinking about strategies that guarantee access to care for this population, considering them as social determinants that directly influence their health.

However, the interviewees also indicated that sex workers often do not seek healthcare services because they believe they will be mistreated or disrespected, which directly results in treatment abandonment or interruption of care. Sex workers, in their work, are understood based on their vulnerability, and it is possible to show that the main markers of social vulnerability of this population are related to socioeconomic data, daily professional routine, social prejudice, fear of seeking healthcare services due to stigma and healthcare professionals who are unqualified to provide care^(1,23)^.

It can be understood that such behavior is a result of the biographical aspects of what was experienced by these people, whose previous experiences of violence and exposure constituted their body of knowledge^(9)^. As an example, the participating nursing team mentions that some cultural aspects increase marginalization and social judgment, including by family members themselves. Sex work is historically linked to society’s moral judgment, which begins with prejudices originating from the family nucleus and extends to healthcare professionals^(24,25)^. Family support and rejection can be listed as aggravating or protective factors, in which negative health outcomes, influenced by rejection and lack of support, lead to suicide attempts, depression and substance use^(24,25)^.

However, few sex workers have social ties with their families, which increases their vulnerability. However, social support and healthcare services can enhance interventions that contribute to reducing vulnerability^(24,25)^. In this context, it is essential that nursing offers care free from personal beliefs, moral values or judgments, prioritizing the construction of trusting relationships and the strengthening of social and institutional support, essential elements to reduce vulnerability, promote adherence to care, minimize negative impacts on health and ensure comprehensive care.

Participants pointed out aspects that characterize part of the lifeworld and biography of sex workers, such as going to BHU accompanied by a henchman, having their personal documents withheld or finding themselves in a situation of imprisonment at the workplace. The conditions reported permeate the health-disease process of sex workers, representing a daily life of absence and violations of basic human needs and minimum rights, such as access to healthcare^(1,3)^.

On the threshold between criminalization and regulation of sex work, experiences such as that of the European Union indicate that, by criminalizing, marginalization and stigmatization increase, and this brings even more risks of health problems, such as exposure to STIs^(26)^. However, structural changes are needed in legislation that guarantees labor rights and policies that address human rights, combating stigma and discrimination^(26)^. From this, it is emphasized that nursing can act in these scenarios by promoting respectful care and mediation in access to health for sex workers through the identification of risk situations and violations, guaranteeing basic rights and raising awareness among managers for inclusive policies that combat stigma, vulnerability and promote comprehensive health.

As a result of the work dynamics experienced by sex workers, there are demands that they bring when they arrive at BHU and are welcomed by the nursing team: from complaints of gynecological conditions that already present a risk to health to the search for support in situations of violence. Thus, the members of the nursing team emphasize in the study that they welcome, listen, provide guidance and promote the health of the sex worker who seeks the unit. We pointed out that these care actions used by the nursing team in accessing sex workers are organized through face-to-face relationships, since it is in the encounter with the other that the care needs are identified, with the possibility of beginning to build a bond^(9)^.

Still regarding the role of nursing in BHU, the work extends beyond the first contact with patients, in which it is recommended that their demands be assessed from an interprofessional perspective and in collective spaces that enhance joint action in care planning^(27)^. Furthermore, with the challenge of monitoring and returning sex workers to BHU, there is a need to carry out an active search to reinforce the connection to the service, carry out an assessment of the general health condition and provide guidance to prevent various problems^(27)^. Therefore, it is recommended that the nursing team participate in spaces for discussing cases and take part in actions that support the link between BHU and sex workers to identify their health needs and expand the scope of care, doing so through active search, home visits and health promotion and prevention actions in the territory.

It is possible to relate that the use of active search as a strategy for returning to BHU makes the marginalization and judgment of sex workers explicit. The lack of regulation in professional practice reinforces the maintenance of vulnerability, invisibility in government public policies, lack of social protagonism, stigmas and occurrence of sexual exploitation^(1,5)^.

It is worth highlighting that the elements of tension originating from the world of work are important for understanding the lifeworld of this population, and call upon the natural attitude, which promotes resistance to care interpreted by the nursing team through abolitionist lens, a reality that requires transformation through investment in face-to-face relationships, which can favor knowledge of the lifeworld of these subjects and openness to change^(9)^. Sometimes, the care provided by the nursing team is directed to the parts of the body that correspond to the nature of sex workers’ work, with this population being understood from the point of view of establishing sexual relations, which limits assistance to sexual and reproductive issues, prevention of STIs and contraception^(5)^.

However, the team also develops actions that contribute to subjectivization of these professionals, placing them as leading actors in health production. This occurs when they base care on face-to-face relationships, based on active listening and knowledge of family backgrounds, understanding the history and offering psychological help^(9)^. Thus, it is possible to indicate that there is a conflict in relation to the natural attitude of the body as an object of intervention in nursing care, when understanding sex workers only from the perspective of their work activity, since, at the same time, there are important care actions that advance in relation to this highlighted understanding, which makes it possible to point to soft technologies as strategies of subjectivation with a potential to transform the lifeworld of sex workers through nursing care.

In face-to-face relationships, the nursing team demonstrates intentionality in caring for sex workers, promoting health, building bonds to ensure their return to work, improving their quality of life and raising awareness about their rights to their bodies. This approach demonstrates motivation to provide effective and transformative care, aligned with this population’s needs and the PHC objective of promoting comprehensive care in the territory^(8,18)^.

The desire for well-being in relation to others is part of the natural attitude of the nursing work process, whose purpose is to develop the action of caring for others, with health going beyond being sick or not, presenting itself as a plural consideration of individuals who establish themselves in the world biopsychosocially^(8)^. Therefore, the nursing team, based on the knowledge acquired throughout their professional training, recognizes the right of sex workers to care based on public policies. By aligning their intentions with this population’s health needs, they demonstrate motivation to develop resolute and transformative nursing actions from this perspective, reaffirming the role of PHC in promoting comprehensive, humanized care centered on the biopsychosocial perspective.

This study highlights the actions and contributions of nursing in PHC, emphasizing its essential role in caring for sex workers, even in the face of the challenges imposed by the social marginalization of this population. The importance of interdisciplinary and multidisciplinary work, building bonds and promoting autonomy is highlighted, even in adverse working conditions and in the face of bureaucratic barriers. Furthermore, nursing can act in the processes of implementing inclusive public policies, proposing strategies that expand access and ensure continuity of care, reinforcing the PHC principles of comprehensiveness and person-centeredness.

As for study limitations, we highlighted that it is a specific territory of a municipality and that the complexity of the studied theme permeates other social determinations for reading the lifeworld that this study did not propose to analyze. It is also necessary to broaden the investigations on the theme addressed, considering the intersection with other social markers of difference and the adoption of varied methodological approaches. This is due to the fact that the phenomenological perspective, by privileging a deep and detailed analysis of the nuances of the experience lived by participants, is characterized by prioritizing data quality and descriptive richness rather than sample size^(15)^. This approach emphasizes that rigor and depth in understanding the phenomenon studied are intrinsically linked to the ability to capture and describe, in detail, participants’ experiences^(15)^.

## CONCLUSION

This study found that nursing team perceptions of the care provided to sex workers consider their actions based on aspects of their life biography and the body of knowledge of their lived experiences.

It was evident that the nursing team’s work in PHC is permeated by the dynamics of the territory in which it operates, marked by the presence of drug trafficking and prostitution, imposing additional challenges to care practice. By recognizing the vulnerabilities faced by sex workers, the need for interdisciplinary action and strategies that promote access and continuity of care stands out, even in the face of the barriers imposed by social marginalization.

The study findings also reveal that nursing staff play a fundamental role in building bonds, using care technologies that seek to overcome bureaucratic barriers. However, precarious working conditions, lack of regulation of professional practice and social invisibility make it difficult for sex workers to seek healthcare. It is recommended that healthcare services develop strategies that facilitate access and the right to health, in addition to more inclusive public policies.

Finally, the face-to-face relationship established between the nursing team and sex workers reveals an ambiguity between the natural attitude as a limitation of care for sexual and reproductive issues and subjectivation promotion, which recognizes these individuals as leading actors of their own health. The motivation of nursing teams to promote autonomy and well-being in the lifeworld of others reflects the essence of PHC as a gateway to SUS, where care must be decisive, comprehensive and person-centered.
