# Supplementary material for: Nursing team perceptions in caring for sex workers in primary care
Source: Rev Esc Enferm USP. 2025 Apr 11;59:e20240331. doi: 10.1590/1980-220X-REEUSP-2024-0331en (PMC11991667; doi:10.1590/1980-220X-REEUSP-2024-0331en)
Supplement: Translation

# Translation: Percepções da equipe de enfermagem no cuidado a profissionais do sexo na atenção primária

**DOI:** 10.1590/1980-220X-REEUSP-2024-0331pt

**Authors:** Helen Prates Rodrigues, Giulia Delfini, Aldair Weber, Ana Paula Rigon Francischetti Garcia, Vanessa Pellegrino Toledo

**Affiliations:** 1 Universidade Estadual de Campinas, Faculdade de Enfermagem, Campinas, São Paulo, SP, Brasil.

**Keywords:** Equipe de Enfermagem, Cuidado de Enfermagem, Profissionais do Sexo, Acesso à Atenção Primária

## Abstract

**Objetivo:**

Compreender a percepção da equipe de enfermagem de Unidade Básica de Saúde de uma cidade do interior de São Paulo sobre os cuidados prestados a profissionais do sexo.

**Método:**

Trata-se de pesquisa qualitativa baseada no referencial de Alfred Schutz, com entrevistas semiestruturadas realizadas junto a 12 profissionais de enfermagem.

**Resultados:**

A categoria “Percepções da equipe de enfermagem e seu mundo vida no cuidado aos profissionais do sexo” desvelou os motivos “porque”, destacando os desafios enfrentados no cuidado de enfermagem em contextos vulneráveis. Já os motivos “para” estão presentes na categoria “A intencionalidade do cuidado prestado”, que evidencia a busca por um cuidado resolutivo e promoção da autonomia no mundo vida dos profissionais do sexo.

**Conclusão:**

A percepção da equipe de enfermagem acerca do cuidado prestado reconhece as necessidades desses profissionais e, por meio do vínculo e acolhimento, busca estratégias que promovam o acesso aos serviços de saúde.

## INTRODUÇÃO

Os profissionais do sexo compreendem uma ampla diversidade de indivíduos, desenvolvendo seu trabalho em distintos locais e contextos socioculturais^(1)^. Globalmente, enfrentam graves desigualdades de saúde e direitos humanos, incluindo exposição à violência, infecções sexualmente transmissíveis (ISTs), além de necessidades de cuidado em saúde sexual e reprodutiva não atendidas devido às fragilidades dos cuidados primários^(1)^.

No Brasil, há cerca de 20 anos, os profissionais do sexo foram oficialmente reconhecidos como uma ocupação pela Classificação Brasileira de Ocupações do Ministério do Trabalho e Emprego^(2)^. Embora a atividade seja legalmente assentida, sua organização e funcionamento dependem inteiramente deles próprios, que permanecem expostos a diversas vulnerabilidades^(3)^.

Este cenário ocorre devido às vivências dos profissionais do sexo dentro do processo de saúde-doença, tornando-os pessoas vulneráveis. No cotidiano, há agravantes dessa dinâmica em decorrência das falhas das ações nos níveis sociais, individuais e programáticos, como o desamparo do Estado, a necessidade de exposição para sobreviver, as condições precárias de moradia, trabalho e o estigma social^(4)^. Dessa forma, a saúde desses profissionais se encontra constantemente em risco, expondo-se a ISTs por meio de relações sexuais sem uso de preservativos, ao abuso de álcool e drogas, ao estresse psicológico causado por violência física, verbal e sexual, além de um ambiente de trabalho prejudicial^(4)^.

Ao buscarem os serviços de saúde, esses profissionais enfrentam situações de violência institucional, encontrando barreiras no acesso aos cuidados ou sendo limitados à abordagem de questões pontuais, como o uso de preservativos e anticoncepcionais. A falta de políticas de saúde direcionadas ao cuidado integral dessa população, juntamente com preconceitos e marginalização social, contribui para uma assistência fragmentada e aumento da inequidade no acesso^(5)^.

Considerando as necessidades de saúde e vulnerabilidades desta população, é garantido o direito de acesso de qualquer pessoa aos serviços do Sistema Único de Saúde (SUS) no Brasil, sendo a Atenção Primária à Saúde (APS) a principal porta de entrada preferencial na Rede de Atenção à Saúde do país, proporcionando um cenário ideal para o cuidado contínuo, longitudinal e centrado nas pessoas^(6)^. O acesso dos usuários ao sistema de saúde depende de fatores organizacionais e geográficos, pois as condições das pessoas e dos serviços influenciam sua utilização e continuidade do cuidado^(7)^.

Neste contexto, a enfermagem apresenta-se como importante protagonista da organização e do funcionamento da APS, pois busca garantir o acesso inicial, o seguimento em saúde, a integralidade nas ações, além da coordenação, orientação familiar e comunitária, e aspectos culturais^(8)^. Esses elementos promovem um vínculo entre profissionais e população, visando oferecer um cuidado acolhedor, eficaz e sem discriminação, baseado em princípios de promoção, prevenção, tratamento e reabilitação das pessoas^(8)^. Assim, destaca-se a enfermagem como precursora da atenção integral aos profissionais do sexo, desde a identificação do território, acolhimento de suas necessidades até a garantia da continuidade dos cuidados de saúde.

Portanto, este estudo justifica-se pela necessidade de promover um cuidado integral para populações vulneráveis e garantir a universalidade do acesso aos serviços de saúde^(4,5)^. Embora os profissionais do sexo sejam oficialmente reconhecidos como ocupação no Brasil, ainda enfrentam múltiplas vulnerabilidades, como exposição a ISTs, violência e condições de trabalho precárias^(4–6)^. A ausência de políticas de saúde direcionadas ao cuidado integral, aliada ao estigma social e preconceito no atendimento, contribui para uma assistência fragmentada e insuficiente^(1,7)^. Nesse contexto, a atuação da enfermagem na APS é importante para construir um cuidado contínuo, humanizado e centrado nas necessidades socioculturais desses profissionais^(8)^. No entanto, persistem lacunas no conhecimento sobre o cuidado a essa população, especialmente quanto à ausência de políticas específicas, à necessidade de identificar e reduzir barreiras de acesso aos serviços de saúde, e à importância de combater a marginalização social, o estigma e o preconceito presentes no atendimento a profissionais do sexo^(1,4–8)^.

Diante desta problemática, a inquietação dos pesquisadores reside na importância de compreender as vivências da equipe de enfermagem que atua em um território marcado pela alta confluência de profissionais do sexo. Tal inquietação ainda é justificada pela consolidação da APS como porta de entrada preferencial do SUS, o que exige das equipes de saúde o exercício de seu protagonismo para uma assistência integral, inclusiva, equânime e sem discriminação no acesso das pessoas a rede de atenção. Este estudo, portanto, busca compreender as percepções da equipe de enfermagem sobre o cuidado dos profissionais do sexo no território de uma Unidade Básica de Saúde (UBS) de um município do interior paulista.

## MÉTODO

### Desenho do Estudo

Trata-se de estudo qualitativo, baseado no referencial teórico-metodológico da fenomenologia de Alfred Schutz, que objetiva desvelar o fenômeno por meio da ação social, compreendida como um ato intencional composto por motivações projetadas pelo indivíduo em seu mundo vida, permeado pelas relações intersubjetivas face a face e sua situação biográfica, conformando seu acervo de conhecimento^(9)^. Foram seguidas as recomendações do *COnsolidated criteria for REporting Qualitative research (COREQ)* ^(10)^.

### Local

O estudo foi desenvolvido em uma UBS de um município do interior paulista, serviço integrante da rede de saúde pública e localizada em uma região criada pelo poder público em 1967, a partir de um processo de higienização urbana que consistiu na retirada de profissionais do sexo e das casas de prostituição do centro da cidade, realocando-os para um espaço afastado. Essa ação visou à separação espacial e geográfica do que era considerado “sujo ou impróprio” para o convívio das famílias^(11)^.

Na configuração urbana atual, o território de abrangência da UBS concentra os estabelecimentos de prostituição, principalmente no bairro Jardim Itatinga, o que define a paisagem do local^(11)^. O bairro se localiza na região Sudoeste do município, cerca de dez quilômetros do centro, em uma área desvalorizada economicamente e que concentra o maior número de moradores de baixa renda^(11)^. É atualmente considerada a maior zona de prostituição a céu aberto da América Latina, com uma circulação aproximada de 2.000 profissionais do sexo por mês, o que permite o funcionamento de inúmeras casas de prostituição de diversos tamanhos^(12)^.

Logo, o território de abrangência da UBS possui características de exclusão, marginalização e estigma da população e dos profissionais do sexo em relação ao restante da cidade, visto que foi escolhido por ter sido, na época, um lugar isolado, pouco urbanizado e de difícil acesso, marcando esse público por uma vulnerabilidade social e moral^(12)^. Atualmente, a UBS situa-se nos arredores da área do território que concentra os estabelecimentos de prostituição, e conta com equipes de Estratégia Saúde da Família para ofertar atendimentos com profissionais da equipe multiprofissional, serviços assistenciais, exames de apoio diagnóstico, vigilância em saúde e dispensação de medicações^(13)^.

Cabe destacar que os pesquisadores integrantes deste estudo desenvolvem suas práticas de pesquisa, ensino e assistência de enfermagem em diversos dispositivos de saúde do município, desde a APS até serviços especializados. Dessa forma, o cuidado indireto e direto aos profissionais do sexo ocorre cotidianamente nesses distintos espaços, trazendo proximidade à temática abordada.

### População

Participaram deste estudo todos os profissionais da equipe de enfermagem de uma UBS, incluindo enfermeiros, técnicos e auxiliares de enfermagem. O acesso aos participantes iniciou-se com a presença da pesquisadora principal em uma reunião de equipe na UBS, onde foi realizada a apresentação do estudo e o convite para participação. Na ocasião, houve a adesão de um profissional, que caracterizou o primeiro participante do estudo e que, subsequentemente, indicou o próximo profissional que, por sua vez, também aceitou participar e indicou o seguinte. Esse processo foi repetido até que todos os membros da equipe de enfermagem tivessem participado da pesquisa.

### Critérios de Seleção

Foram incluídos membros da equipe de enfermagem e que tinham a vivência de cuidado dos profissionais do sexo no território estudado. Foram excluídos profissionais em período de afastamento e férias.

### Coleta de Dados

A coleta de dados foi realizada nos meses de abril e maio de 2021, utilizando a entrevista fenomenológica, que propicia ao sujeito que vivencia o fenômeno compartilhar com o entrevistador o significado da ação desenvolvida em seu mundo de relações intersubjetivas^(14)^. As questões disparadoras foram “Você já cuidou de algum profissional do sexo nesse serviço?”, “Conte-me como foi” e “Qual sua intenção ao realizar esse cuidado?”, e, a partir destas, foram sendo elaboradas outras questões que permitiram ao entrevistado aprofundar a temática.

As entrevistas ocorreram em local privativo nas dependências da UBS, sendo conduzidas pela pesquisadora principal, gravadas em áudio digital, com duração média de 15 minutos, sem nenhuma recusa pelos participantes. A coleta de dados foi encerrada quando a pesquisadora compreendeu que o fenômeno foi desvelado e, sua inquietação, sanada, e nenhum elemento novo foi identificado na constituição das categorias que representam o tipo vivido da experiência^(15)^. A inserção da equipe de pesquisa na UBS ocorreu durante a coleta de dados e, posteriormente, na devolutiva ao serviço sobre o estudo desenvolvido.

### Análise e Tratamento dos Dados

A partir do referencial teórico da fenomenologia social^(9)^, os dados foram analisados e categorizados pelos pesquisadores que contribuíram para o estudo, sem uso de *software*. Foram analisadas todas as entrevistas realizadas. Observaram-se os passos indicados em estudos fundamentados nos conceitos de Alfred Schutz^(9)^: transcrição na íntegra dos depoimentos dos participantes; leitura e releitura cuidadosa das entrevistas para compreender o significado da vivência dos profissionais da equipe de enfermagem; apuração dos trechos que incluem sentidos comuns das vivências e experiências dos participantes; e organização dos discursos em categorias temáticas que demonstram os “motivos por que” e “motivos para” do vivido pelos entrevistados^(9)^. Utilizaram-se a fenomenologia social e a literatura científica do tema abordado para realizar análise compreensiva e discussão dos resultados apresentados nas categorias.

### Aspectos Éticos

A pesquisa atendeu a todos os critérios da Resolução n^o^ 466/12, que regulamenta pesquisas envolvendo seres humanos, e foi aprovada pelo Comitê de Ética em Pesquisa da Universidade Estadual de Campinas, sob Parecer nº 4.659.425. Os participantes da pesquisa assinaram o Termo de Consentimento Livre e Esclarecido e o Termo de Autorização para Gravação da Voz antes do início das entrevistas. Para garantir o anonimato aos participantes, os depoimentos foram identificados pela letra “E” para representar a palavra “entrevistado”, seguida do número correspondente à ordem cronológica em que as entrevistas se sucederam.

## RESULTADOS

O estudo foi composto por 12 participantes, sendo três enfermeiros e nove técnicos de enfermagem, com média de dois anos de atuação dos enfermeiros na unidade e de sete anos para os técnicos de enfermagem. As entrevistas possibilitaram a construção da relação “nós”, organizando as vivências do outro em duas categorias: a primeira evidenciou os “motivos por que” do mundo vida dos participantes, nomeada como “Percepção da equipe de enfermagem sobre o território e o serviço de saúde”; e os motivos para ação dos participantes estão descritos na categoria “A intencionalidade do cuidado prestado”.

### Percepção da Equipe de Enfermagem Sobre o Território e o Serviço de Saúde

Durante as entrevistas, foram relatadas características do território, o qual corresponde a uma área de prostituição e tráfico, resultando em um afastamento do profissional na abordagem e no envolvimento. O território determina problemas de saúde decorrentes do uso e comercialização de substâncias psicoativas e álcool. Há também o reconhecimento de que a população deste território é marcada pela vulnerabilidade e dificuldade da manutenção do vínculo.

*Não é só prostituição que tem aqui, tem o tráfico também. Então, tem coisa que você não pode se envolver, que daí a gente corre risco de vida... a gente tá em um território que tem que ter muito cuidado com o que a gente fala, o que a gente faz, a abordagem*. (E3)

*E a gente sabe que às vezes a pessoa chega sem um problema específico, ou não usa droga, não é usuário de droga e álcool e para conseguir levar a vida se envolve. Se envolve em um nível que vai para o tráfico, que vai muito além do que aparenta*. (E10)

*Acho que a rotina é igual a um centro de saúde qualquer, só que o único diferencial é que a nossa população é uma população mais exposta e mais vulnerável*. (E12)

A equipe de enfermagem reconheceu que a atuação junto aos profissionais do sexo era maior quando a UBS estava inserida na área de prostituição. A proximidade do serviço junto à população favorecia maior cuidado diário, mas agora não há cuidado de prevenção à saúde, e a busca por atendimento é realizada quando há queixa.

*Antigamente, quando a gente estava dentro do* [bairro]*, a gente tinha uma atuação muito maior, porque eles vinham, tinham uma demanda, a gente estava dentro da prostituição, a gente estava dentro da zona, e hoje não*. (E4)

*O acesso ao cuidado diário era maior. Agora não, agora elas só nos procuram quando realmente estão com uma queixa. Não tem cuidado preventivo, então acho que fez uma diferença essa nossa saída do território. Apesar de estarmos ainda, mas pra elas, fisicamente, isso mudou*. (E7)

Quanto às características do serviço, observou-se que o atendimento é realizado sem a necessidade de apresentação de documento de identificação, o que é justificado pela valorização do cuidado à saúde da pessoa. O serviço estrutura o atendimento a partir da demanda espontânea e do acolhimento, buscando atender ao máximo as demandas para favorecer o vínculo e o retorno ambulatorial.

*Elas vêm sem a identidade para ser atendida... tem várias questões que, se a gente não tiver um jogo de cintura, tentar trabalhar mesmo sem a identidade, porque eu tô cuidando da pessoa, então... eu sei, se eu ver, eu sei quem é, mas pode ser que ela mentiu o nome também. Então, a gente toma muito cuidado com essa questão do atendimento também. Mesmo que tá sem a identidade, a gente atende, faz o cuidado mesmo de saúde.* (E3)

*Essas mulheres normalmente não vêm para atendimento programático, elas vêm sempre por demanda espontânea no acolhimento. No geral, é uma população que não vale a pena agendar. A maioria das profissionais são de outros municípios, então vai dar seguimento aqui com a gente mesmo. Então, a gente tenta atender o máximo possível a demanda que ela traz no dia, pra tentar vincular e trazer ela para o ambulatório.* (E1)

A equipe de enfermagem elenca que os profissionais do sexo possuem fragilidades em sua saúde, devido à exposição a ISTs, problemas de saúde mental, histórico de abusos e uso de substâncias psicoativas. Refere também a não procura do serviço pelos profissionais do sexo por acharem que serão maltratados, e, quando chegam, abandonam o tratamento ou não esperam o desfecho da atenção oferecida.

*É frágil de saúde, né, pela exposição de IST, frágil pela saúde mental, geralmente são pessoas que foram abusadas. Tem muita história de abuso nesse meio, porque, como eu disse, pra sustentar essa vida noturna, é bem complicado. Então, eles se veem bem perdidos, e o caminho é retornar às drogas ou ao álcool.* (E10)

*Muitas delas nem vem porque acham que vão ser maltratadas... elas mesmas criam aquela casca protetora. Muitas delas acabam por abandonar o tratamento. A gente vai atrás*. (E11)

*Por elas terem muita pressa, às vezes, enquanto a gente tá rodando o teste rápido, quando a gente chama pra dar o resultado, elas já foram embora.* (E1)

Quanto aos aspectos culturais, a equipe de enfermagem identificou que os profissionais do sexo são marginalizados e julgados pela família. Identificam em si também uma bagagem de preconceitos provinda do núcleo familiar. Apontam que os profissionais de saúde possuem estigmas que resultam na recusa em atender determinados grupos.

*Eles são muito marginalizados, né, são julgados demais, até pela própria família*. (E10)

*Independente de ser profissional do sexo ou não, a gente vem com muita bagagem da nossa família; às vezes, tem um preconceito.* (E3)

*A gente tem esse estigma de separar as pessoas pelo que ela aparenta, pelo que ela é, porque eu já trabalhei em várias unidades e muito tem esse negócio “Ai, eu não atendo paciente de saúde mental, eu não atendo homossexual”. Porque não é só o auxiliar de enfermagem, o técnico de enfermagem que precisa de capacitação, porque já observei profissional de nível superior, médico, fazendo essa separação.* (E6)

Quanto às características de trabalho dos profissionais do sexo identificados pela equipe de enfermagem, incluem-se a ida ao serviço acompanhados de “capanga”, limitando sua permanência no local, e a retenção da identidade pela casa em que trabalham. Ainda, relatam condições de trabalho caracterizadas pelo cárcere e pagamento de multas durante a ausência.

*Normalmente, elas vêm com o capanga da casa junto. Então, elas têm um tempo que elas podem ficar aqui para serem atendidas. Então, se eu demoro, tô com urgência, elas não esperam, elas vão embora. Tem algumas meninas que a identidade fica retida na casa.* (E3)

*Elas são submetidas às mais diversas condições de trabalho; às vezes, ficam em cárcere. Até porque o tempo em que elas ficam fora, elas têm que pagar multa por não tá na casa ou vem alguém para fazer esse monitoramento.* (E1)

Quanto às demandas dos profissionais do sexo, observou-se que as queixas estão relacionadas a problemas ginecológicos que possam impedir a continuidade do trabalho, quadros infecciosos, busca por medicação, violência, acidentes com preservativos, procura por testes rápidos e gravidez indesejada.

*Elas vêm procurar a gente por questões relacionadas ao problema ginecológico ou problema de violência ou acidente com preservativo.* (E12)

*As prostitutas chegavam bem machucadas pra gente; às vezes, eram jogadas de carro, né... elas chegavam todas esfoladas e a gente cuidava*. (E8)

*Essa é a entrada, porque a exposição foi, eu vou tomar a medicação, mas a porta principal é sim o teste rápido.* (E10)

*Elas sempre vêm. Às vezes, vêm com uma gestação que não quer.* (E2)

*Às vezes, tá com dois dias, tá com cheiro mal, tem que entrar com antibiótico porque já tá com dor, já tá com o processo infeccioso ali.* [...] *e outra coisa é que, no período menstrual, elas usam algodão dentro do fundo vaginal pra poder ter relação. Então, é comum chegar a menina que não conseguiu tirar o algodão, pra gente tirar.* (E3)

A equipe de enfermagem reconhece fazer da melhor maneira possível e sem descriminação o trabalho de acolhimento, escuta, antecedentes de saúde pessoais e familiares, com resolução das demandas e queixas levadas pelos profissionais do sexo, promovendo saúde e orientando em relação aos riscos. A equipe também realiza abordagem para entender o histórico do paciente, o motivo da procura da unidade e se precisam de alguma ajuda psicológica.

*Meu trabalho é acolher, escutar e tentar resolver de forma conjunta com elas, dando uma solução pra aquilo, das demandas que elas me trazem.* [...] *a gente tem que acolher, escutar e tentar promover saúde pra eles. Orientar em relação aos riscos, e é isso.* (E2)

*Dentro daquilo que eles nos procuram, a gente atende, tenta atender da melhor maneira possível, sem fazer discriminação nenhuma, né?* (E9)

*Você vê o paciente, questão de saúde de antecedentes familiares, o que ele já teve, qual tipo de doenças, você pergunta sobre vacina, você faz tudo, e depois você vai para a parte da queixa principal do paciente.* (E12)

*Primeiro, a gente faz a abordagem, tenta entender um pouco da história, porque tá procurando o serviço.* [...] *então, tentar entender como é isso pra ela. Se tá tudo bem, se precisa de alguma ajuda psicológica.* (E3)

Os profissionais realizam procedimentos de acordo com a queixa trazida pelo paciente, fornecem orientações diversas e realizam busca ativa para a continuidade e término do tratamento.

*A gente coloca espéculo, tira o algodão, orienta do risco, orienta do coletor menstrual. Assim, são as orientações mais diversas e ginecológicas.* (E1)

*Problemas com os testes, que a gente faz dos DSTs, a gente cuida dos DSTs, instrui.* [...] *como eu falei, tem que ir atrás, porque a maioria não volta. Então, a gente que fica atrás, tem que ficar em cima para elas terminarem o tratamento*. (E11)

### A Intencionalidade do Cuidado Prestado

Quanto à expectativa do cuidado prestado, a equipe de enfermagem espera promover saúde para profissionais do sexo, diminuir o risco de transmissão de doenças, acolhê-los, que retornem para os atendimentos, tenham sucesso e qualidade de vida. Também esperam que o cuidado seja resolutivo e faça diferença na vida daquela pessoa. Por fim, almejam a melhora do cuidado e proteção, a diminuição da exposição e que entendam a necessidade e direito do cuidado ao corpo.

*Espero que promova o bem, promova a saúde pra elas.* (E2)

*Diminuir o risco de transmissão de doenças... eles se sentirem também acolhidos* [...] *e retorno, né, pedir retorno, eles ter onde procurar o que eles precisam.* (E5)

*Sucesso! Assim, que eles consigam ter uma qualidade de vida.* (E10)

*Espero que seja resolutivo e espero que faça diferença na vida daquela pessoa.* (E1)

*A gente espera que elas consigam se cuidar de uma maneira melhor, consigam se proteger de uma maneira melhor, não se expondo tanto.* [...] *mas é isso, ela tentar entender que ela tem que cuidar do corpo dela. Mesmo ela sendo profissional, ela tem o direito ao cuidado do corpo dela.* (E12)

## DISCUSSÃO

Os profissionais de enfermagem participantes do estudo relataram suas vivências e experiências no cuidado aos profissionais do sexo no território de abrangência da UBS, com a demarcação da presença do tráfico de drogas e da prostituição no local de abrangência, o que influencia como será a atitude da equipe de enfermagem ao cuidar dessa população. Toda ação traz uma expectativa de resposta a esta, e neste cenário, é possível observar o território como o espaço de encontro subjetivo do mundo vida do profissional e das pessoas que nele existem, tal como os profissionais do sexo^(9)^.

O território é dinâmico, e a partir das relações criadas, pode ser produto ou produtor de ações. Assim, na leitura da realidade apresentada ao indivíduo, convida-se este a apresentar a forma como se coloca no território, denominada atitude natural^(9)^. Por meio desta, é possível a abordagem do cuidado em locais que possam causar tensão e medo aos profissionais de saúde, devido à presença da violência^(16)^.

Toma-se por território na APS um espaço geográfico delimitado, com uma população adscrita, dinâmico e em constante mudança, sujeito a múltiplas variáveis, tal como as vulnerabilidades sociais^(16)^. Nesse espaço, ocorre a produção do processo de saúde-doença, resultado dos determinantes sociais da saúde, caracterizados como fatores relacionados às condições de vida e trabalho de um indivíduo, podendo influenciar o surgimento de agravos à saúde^(16,17)^. Dessa forma, o território da UBS deste estudo se apresenta como resultado de uma dinâmica histórica em constante movimento que requer seu reconhecimento para realizar o cuidado em saúde, pois sua origem remonta à segregação social e às estratégias de higienização de grandes centros urbanos.

Contudo, os profissionais de saúde da UBS relataram dificuldades para atuar nesse cenário, como as barreiras para acessar a população, manter-se presente no território e sustentar vínculos. O território adscrito da APS pode se deparar com outras distintas divisões de organizações de pessoas, como o tráfico de drogas, porém é importante considerar que a população que ali reside apresenta necessidades de saúde e cuidado, o que requer o reconhecimento e planejamento de ações a serem desenvolvidas^(16)^.

É mencionado pelos participantes que a UBS já se localizou entremeio às casas de prostituição, o que representava uma estratégia de acesso e proximidade da população em relação aos serviços de saúde. Porém, na organização atual, o cuidado é produzido somente por meio da queixa, pois requer o deslocamento dos profissionais do sexo até o serviço de saúde. Recomenda-se que a localização da UBS deva considerar os aspectos geográficos para facilitar o acesso da população, o que muitas vezes não se torna possível, e as unidades acabam alocadas em espaços de difícil acesso^(18)^.

Assim, cabe aos profissionais de enfermagem colaborar na construção de estratégias que diminuam as barreiras de acesso às populações vulneráveis, iniciando pelo reconhecimento do território de abrangência do serviço de saúde até a identificação das pessoas que ali residem. Os participantes do estudo mencionam ações de acolhimento a partir da busca por demanda espontânea e não imposição da apresentação de documentos de identificação como formas de acolher e garantir o acesso aos profissionais do sexo na UBS. Ressalta-se que assegurar essas estratégias é essencial para garantir o direito das pessoas ao SUS, resultado de investimentos no processo de trabalho das equipes multiprofissionais, cuja presença ativa dos profissionais é indispensável, pois, juntos, buscam estabelecer uma relação de confiança com os usuários, promovendo a construção de soluções e formas de cuidado^(19)^.

Além disso, os participantes apontaram que as necessidades de saúde dos profissionais do sexo são permeadas pela exposição às ISTs, uso abusivo de substâncias psicoativas, violência e problemas relacionados à saúde mental. Estudos apontam que os agravos de saúde mental dos profissionais do sexo são frequentemente mediados pelo local onde trabalham e por sua vulnerabilidade à violência, ao abuso de substâncias e ao estigma^(1,20)^. As ISTs somam-se como fator agravante à saúde mental desta população, pois trata-se de fenômeno complexo e multifatorial, cujas principais medidas estão direcionadas à prevenção, detecção precoce e tratamento oportuno, potencializadas pelo acesso aos serviços de saúde^(21,22)^. Todos esses eventos associados e vividos pelos profissionais do sexo constituem sua biografia de vida, aspecto importante para a equipe de enfermagem ao pensar estratégias que garantam acesso ao cuidado dessa população, considerando-os como determinantes sociais que influenciam diretamente sua saúde.

Entretanto, também é sinalizado pelos entrevistados que, muitas vezes, os profissionais do sexo não buscam o serviço de saúde por acharem que serão maltratados ou desrespeitados, cujo resultado direto é o abandono do tratamento ou interrupção do cuidado. Os profissionais do sexo, em seu exercício laboral, são compreendidos a partir de sua vulnerabilidade, sendo possível evidenciar que os principais marcadores de vulnerabilidade social dessa população estão relacionados aos dados socioeconômicos, cotidiano da profissão, preconceito social, medo de procurar os serviços de saúde devido ao estigma e profissionais de saúde desqualificados para o atendimento^(1,23)^.

Pode-se compreender que tal comportamento é resultante dos aspectos biográficos do que foi vivido por essas pessoas, cujas experiências anteriores de violência e exposição constituíram seu acervo de conhecimento^(9)^. Como exemplo, a equipe de enfermagem participante cita que alguns aspectos culturais potencializam a marginalização e o julgamento social, inclusive pelos próprios familiares. O trabalho sexual está historicamente atrelado ao julgamento moral da sociedade, que se inicia com preconceitos advindos do núcleo familiar e se estendem até os profissionais de saúde^(24,25)^. O apoio e a rejeição familiar podem ser elencados como fatores agravantes ou protetivos, em que resultados negativos de saúde, influenciados pela rejeição e falta de suporte, suscitam em tentativas de suicídio, depressão e uso de substâncias^(24,25)^.

No entanto, poucos profissionais do sexo têm laços sociais com sua família, o que intensifica sua vulnerabilidade. Contudo, o apoio social e de serviços de saúde pode potencializar intervenções que contribuam na diminuição da vulnerabilidade^(24,25)^. Nesse contexto, é fundamental que a enfermagem ofereça um cuidado livre de crenças pessoais, valores morais ou julgamentos, priorizando a construção de relações de confiança e o fortalecimento do suporte social e institucional, elementos essenciais para reduzir a vulnerabilidade, favorecer a adesão ao cuidado, minimizar os impactos negativos à saúde e assegurar a integralidade do atendimento.

Foram apontados pelos participantes aspectos que caracterizam parte do mundo vida e da biografia dos profissionais do sexo, tais como ir até a UBS acompanhada por um capanga, ter seus documentos pessoais retidos ou encontrar-se em situação de cárcere no local de trabalho. As condições relatadas permeiam o processo de saúde-doença dos profissionais do sexo, representando um cotidiano de ausência e violações das necessidades humanas básicas e de direitos mínimos, como acesso à saúde^(1,3)^.

No limiar entre a criminalização e a regulamentação do trabalho sexual, experiências como a da União Europeia indicam que, ao criminalizar, aumentam-se a marginalização e estigmatização, e isto traz ainda mais riscos de agravos à saúde, como exposição a ISTs^(26)^. Entretanto, são necessárias alterações estruturais em legislações que garantam direitos trabalhistas e políticas que abordem os direitos humanos, o combate ao estigma e a discriminação^(26)^. A partir disso, enfatiza-se que a enfermagem pode atuar nesses cenários promovendo cuidado respeitoso e mediação no acesso à saúde dos profissionais do sexo por meio da identificação das situações de risco e de violações, garantindo direitos básicos e sensibilizando gestores para políticas inclusivas que combatam o estigma, a vulnerabilidade e promovam saúde integral.

Resultante da dinâmica de trabalho vivida pelos profissionais do sexo, encontram-se as demandas trazidas quando chegam à UBS e são acolhidos pela equipe de enfermagem: desde queixas de quadros ginecológicos já apresentando risco para a saúde até a busca por apoio para situações de violência. Assim, os integrantes da equipe de enfermagem destacam no estudo que realizam o acolhimento, a escuta, a orientação e a promoção da saúde do profissional do sexo que busca a unidade. Apontamos que essas ações de cuidado utilizadas pela equipe de enfermagem no acesso aos profissionais do sexo se organizam por meio da relação face a face, pois é no encontro com o outro que se identificam as necessidades de cuidado, com a possibilidade de iniciar a construção de vínculo^(9)^.

Ainda no que tange ao papel da enfermagem na UBS, o trabalho estende-se para além do primeiro contato com o paciente, em que se preconiza que suas demandas sejam apreciadas em uma perspectiva interprofissional e em espaços coletivos que potencializem a ação conjunta no planejamento do cuidado^(27)^. Além disso, com o desafio do seguimento e retorno do profissional do sexo à UBS, há a necessidade de realizar busca ativa para reforçar a vinculação ao serviço, realizar a avaliação do quadro geral de saúde e orientar para prevenir agravos diversos^(27)^. Dessa forma, recomenda-se que a equipe de enfermagem participe dos espaços para discussão de casos e protagonize ações que sustentem a vinculação da UBS com os profissionais do sexo para identificar suas necessidades de saúde e ampliar o alcance do cuidado, fazendo-o por meio de busca ativa, visitas domiciliares e ações de promoção e prevenção em saúde no território.

É possível relacionar que a utilização da busca ativa como estratégia de retorno à UBS explicite a marginalização e o julgamento dos profissionais do sexo. A falta de regulamentação na prática profissional reforça a manutenção da vulnerabilidade, invisibilidade nas políticas públicas governamentais, ausência do protagonismo social, estigmas e ocorrência de exploração sexual^(1,5)^.

Destaca-se que os elementos de tensão originários do mundo do trabalho são importantes para o entendimento do mundo vida dessa população, e convocam a atitude natural, que promove a resistência ao cuidado interpretada pela equipe de enfermagem pela lente abolicionista, realidade que necessita de transformação a partir do investimento da relação face a face, podendo favorecer o conhecimento do mundo vida desses sujeitos e abertura para mudanças^(9)^. Por vezes, o cuidado realizado pela equipe de enfermagem é direcionado às partes do corpo que correspondem à natureza do trabalho dos profissionais do sexo, sendo essa população entendida a partir do estabelecimento das relações sexuais, o que limita a assistência às questões sexuais e reprodutivas, prevenção de ISTs e contracepção^(5)^.

No entanto, a equipe também desenvolve ações que contribuem para a subjetivação desses profissionais, colocando-os como protagonistas na produção de saúde. Isso ocorre quando fundamentam o cuidado na relação face a face, a partir da escuta ativa e conhecimento de antecedentes familiares, entendendo o histórico e oferecendo ajuda psicológica^(9)^. Assim, é possível indicar que existe um conflito em relação à atitude natural do corpo como objeto de intervenção do cuidado de enfermagem, ao compreender os profissionais do sexo somente a partir de sua atividade laboral, pois, ao mesmo tempo, há importantes ações de cuidado que avançam em relação a esta compreensão destacada, o que possibilita apontar para as tecnologias leves como estratégias de subjetivação com um potencial transformador do mundo vida dos profissionais do sexo por meio dos cuidados de enfermagem.

Na relação face a face, a equipe de enfermagem apresenta intencionalidades na ação do cuidado com os profissionais do sexo, promovendo saúde, construindo vínculos para assegurar o retorno ao serviço, melhorando a qualidade de vida e sensibilizando sobre seus direitos ao corpo. Essa abordagem demonstra motivação para oferecer um cuidado resolutivo e transformador, alinhado às necessidades de saúde dessa população e ao objetivo da APS de promover cuidado integral no território^(8,18)^.

O desejo do bem-estar em relação ao outro faz parte da atitude natural do processo de trabalho da enfermagem, cujo propósito é desenvolver a ação de cuidar do outro, sendo que a saúde vai além do estar enfermo ou não, apresentando-se como uma consideração plural do indivíduo que se estabelece no mundo de maneira biopsicossocial^(8)^. Portanto, a equipe de enfermagem, fundamentada em seu acervo de conhecimento construído ao longo de sua formação profissional, reconhece o direito dos profissionais do sexo a um cuidado baseado em políticas públicas. Alinhando suas intencionalidades às necessidades de saúde dessa população, demonstra motivação para desenvolver ações de enfermagem resolutivas e transformadoras dessa perspectiva, reafirmando o papel da APS na promoção de um cuidado integral, humanizado e centrado na perspectiva biopsicossocial.

Este estudo destaca as ações e contribuições da enfermagem na APS, ressaltando seu papel essencial no cuidado a profissionais do sexo, mesmo diante dos desafios impostos pela marginalização social dessa população. Evidencia-se a importância de uma atuação interdisciplinar e multiprofissional, da construção de vínculos e da promoção da autonomia, mesmo em condições de trabalho adversas e diante de barreiras burocráticas. Além disso, a enfermagem pode atuar nos processos de implementação de políticas públicas inclusivas, propondo estratégias que ampliem o acesso e garantam a continuidade do cuidado, reforçando os princípios da APS de integralidade e centralidade na pessoa.

Em relação às limitações do estudo, destacamos tratar-se de território específico de um município e que a complexidade do tema estudado perpassa outras determinações sociais para leitura do mundo vida que este estudo não se propôs a analisar. Observa-se também a necessidade de ampliar as investigações sobre a temática abordada, considerando a interseção com outros marcadores sociais da diferença e a adoção de abordagens metodológicas variadas. Isso se deve ao fato de que a perspectiva fenomenológica, ao privilegiar uma análise profunda e detalhada das nuances da experiência vivida pelos participantes, caracteriza-se por priorizar a qualidade e a riqueza descritiva dos dados em detrimento do tamanho amostral^(15)^. Essa abordagem enfatiza que o rigor e a profundidade na compreensão do fenômeno estudado estão intrinsecamente ligados à capacidade de captar e descrever, de forma detalhada, a vivência dos participantes^(15)^.

## CONCLUSÃO

Este estudo apreendeu que a percepção da equipe de enfermagem acerca do cuidado prestado aos profissionais do sexo considera suas ações a partir de aspectos da biografia de vida e do acervo de conhecimento das experiências vividas.

Evidenciou-se que a atuação da equipe de enfermagem na APS é permeada pela dinâmica do território em que está inserida, marcada pela presença do tráfico de drogas e da prostituição, impondo desafios adicionais à prática do cuidado. Ao reconhecer as vulnerabilidades enfrentadas pelos profissionais do sexo, destaca-se a necessidade de uma atuação interdisciplinar e de estratégias que promovam o acesso e a continuidade do cuidado, mesmo diante das barreiras impostas pela marginalização social.

Os achados do estudo também revelam que a equipe de enfermagem desempenha papel fundamental na construção de vínculos, utilizando tecnologias de cuidado que buscam transpor barreiras burocráticas. Contudo, a precarização das condições de trabalho, a falta de regulamentação da prática profissional e a invisibilidade social dificultam a busca dos profissionais do sexo por cuidados de saúde. É recomendado que os serviços de saúde construam estratégias que facilitem o acesso e o direito à saúde, além de políticas públicas mais inclusivas.

Por fim, a relação face a face estabelecida entre a equipe de enfermagem e os profissionais do sexo revela uma ambiguidade entre a atitude natural como limitadora do cuidado às questões sexuais e reprodutivas e a promoção da subjetivação, que reconhece esses indivíduos como protagonistas de sua saúde. A motivação da equipe de enfermagem em promover autonomia e bem-estar no mundo vida do outro reflete a essência da APS como porta de entrada no SUS, em que o cuidado deve ser resolutivo, abrangente e centrado na pessoa.

# Translation: Percepciones del equipo de enfermería en el cuidado de trabajadoras sexuales en atención primaria

**DOI:** 10.1590/1980-220X-REEUSP-2024-0331es

**Authors:** Helen Prates Rodrigues, Giulia Delfini, Aldair Weber, Ana Paula Rigon Francischetti Garcia, Vanessa Pellegrino Toledo

**Affiliations:** 1 Universidade Estadual de Campinas, Faculdade de Enfermagem, Campinas, São Paulo, SP, Brasil.

**Keywords:** Grupo de Enfermería, Atención de Enfermería, Trabajadores Sexuales, Acceso a Atención Primaria

## Abstract

**Objetivo:**

Comprender la percepción del equipo de enfermería de una Unidad Básica de Salud de una ciudad del interior de São Paulo sobre la atención brindada a las trabajadoras sexuales.

**Método:**

Se trata de una investigación cualitativa basada en el marco de Alfred Schutz, con entrevistas semiestructuradas realizadas a 12 profesionales de enfermería.

**Resultados:**

La categoría “Percepciones del equipo de enfermería y su mundo de vida en el cuidado de trabajadoras sexuales” reveló los “por qué”, destacando los desafíos enfrentados en el cuidado de enfermería en contextos vulnerables. Los motivos “para” están presentes en la categoría “La intencionalidad del cuidado brindado”, que destaca la búsqueda de un cuidado resolutivo y la promoción de la autonomía en el mundo de vida de las trabajadoras sexuales.

**Conclusión:**

La percepción del equipo de enfermería sobre los cuidados brindados reconoce las necesidades de estos profesionales y, a través de la vinculación y la aceptación, busca estrategias que promuevan el acceso a los servicios de salud.

## INTRODUCCIÓN

Los profesionales del sexo comprenden una gran diversidad de individuos, que realizan su trabajo en diferentes lugares y contextos socioculturales^(1)^. A nivel mundial, se enfrentan a graves desigualdades en materia de salud y derechos humanos, como la exposición a la violencia, las infecciones de transmisión sexual (ITS) y las necesidades de atención de salud sexual y reproductiva insatisfechas debido a las deficiencias de la atención primaria^(1)^.

En Brasil, hace unos 20 años, las trabajadoras del sexo fueron reconocidas oficialmente como ocupación por la Clasificación Brasileña de Ocupaciones del Ministerio de Trabajo y Empleo^(2)^. Aunque la actividad esté legalmente reconocida, su organización y funcionamiento dependen enteramente de ellas, que permanecen expuestas a diversas vulnerabilidades^(3)^.

Este escenario se debe a las vivencias de las trabajadoras sexuales dentro del proceso salud-enfermedad, convirtiéndolas en personas vulnerables. En la vida cotidiana, esta dinámica se ve agravada por el fracaso de las acciones a nivel social, individual y programático, como la impotencia del Estado, la necesidad de exponerse para sobrevivir, la precariedad de las condiciones de vivienda y de trabajo y la estigmatización social^(4)^. Como consecuencia, la salud de estos profesionales está en constante riesgo, exponiéndose a las ITS por relaciones sexuales sin uso de preservativo, al abuso de alcohol y drogas, al estrés psicológico causado por la violencia física, verbal y sexual, así como a un entorno laboral nocivo^(4)^. Cuando acuden a los servicios de salud, estas profesionales se enfrentan a situaciones de violencia institucional, encontrando barreras para acceder a la atención o limitándose a tratar temas específicos, como el uso de preservativos y anticonceptivos. La falta de políticas sanitarias dirigidas a la atención integral de esta población, junto con los prejuicios y la marginación social, contribuyen a fragmentar la atención y a aumentar la desigualdad en el acceso^(5)^.

Considerando las necesidades de salud y las vulnerabilidades de esta población, en Brasil cualquier persona tiene garantizado el derecho de acceso a los servicios del Sistema Único de Salud (SUS), siendo la Atención Primaria de Salud (APS) la principal puerta de entrada preferencial a la Red de Salud del país, proporcionando un escenario ideal para la atención continua, longitudinal y centrada en las personas^(6)^. El acceso de los usuarios al sistema de salud depende de factores organizativos y geográficos, ya que las condiciones de las personas y de los servicios influyen en su utilización y en la continuidad de los cuidados^(7)^.

En este contexto, la enfermería es un actor importante en la organización y funcionamiento de la APS, ya que busca garantizar el acceso inicial, el seguimiento sanitario, las actuaciones integrales, así como la coordinación, la orientación familiar y comunitaria y los aspectos culturales^(8)^. Estos elementos promueven un vínculo entre los profesionales y la población, con el objetivo de ofrecer cuidados acogedores, eficaces y sin discriminación, basados en los principios de promoción, prevención, tratamiento y rehabilitación de las personas^(8)^. Así, la enfermería se destaca como precursora de la atención integral a las trabajadoras sexuales, desde la identificación del territorio, la acogida de sus necesidades y la continuidad de los cuidados de salud.

Por lo tanto, este estudio se justifica por la necesidad de promover la atención integral a las poblaciones vulnerables y garantizar el acceso universal a los servicios de salud^(4,5)^. A pesar de que las trabajadoras sexuales son reconocidas oficialmente como una ocupación en Brasil, todavía se enfrentan a múltiples vulnerabilidades, como la exposición a las ITS, la violencia y las precarias condiciones de trabajo^(4–6)^. La falta de políticas de salud dirigidas a la atención integral, combinada con el estigma social y los prejuicios en el servicio, contribuyen a una atención fragmentada e insuficiente^(1,7)^. En este contexto, el papel de la enfermería en la APS es importante para construir cuidados continuos, humanizados y centrados en las necesidades socioculturales de estos profesionales^(8)^. Sin embargo, aún existen lagunas en el conocimiento sobre la atención a esta población, especialmente en lo que se refiere a la falta de políticas específicas, a la necesidad de identificar y reducir las barreras de acceso a los servicios de salud y a la importancia de combatir la marginalización social, el estigma y los prejuicios presentes en la atención a las trabajadoras sexuales^(1,4–8)^.

Frente a esta problemática, la preocupación de los investigadores radica en la importancia de comprender las experiencias del equipo de enfermería que actúa en un territorio marcado por una alta concentración de trabajadoras sexuales. Esta preocupación se justifica, además, por la consolidación de la APS como puerta de entrada preferencial al SUS, lo que exige que los equipos de salud tengan un papel protagónico en la prestación de una atención integral, inclusiva, equitativa y no discriminatoria. Este estudio, por lo tanto, busca conocer las percepciones del equipo de enfermería sobre la atención a trabajadoras sexuales en el territorio de una Unidad Básica de Salud (UBS) de un municipio del interior de São Paulo.

## MÉTODO

### Diseño del Estudio

Se trata de un estudio cualitativo, basado en el marco teórico-metodológico de la fenomenología de Alfred Schutz, que tiene como objetivo develar el fenómeno a través de la acción social, entendida como un acto intencional compuesto por motivaciones proyectadas por el individuo en su mundo de vida, permeado por las relaciones intersubjetivas cara a cara y su situación biográfica, conformando su cuerpo de conocimiento^(9)^. Se siguieron las recomendaciones del*COnsolidated criteria for REporting Qualitative research (COREQ)* ^(10)^.

### Lugar de la Investigación

El estudio se realizó en una UBS de un municipio del interior de São Paulo, servicio que forma parte de la red pública de salud y está localizado en una región creada por el gobierno en 1967 como parte de un proceso de higiene urbana que consistió en retirar a las trabajadoras sexuales y las casas de prostitución del centro de la ciudad y reubicarlas en una zona alejada. Esta acción pretendía separar espacial y geográficamente lo que se consideraba «sucio o no apto» para la vida familiar^(11)^. En la configuración urbana actual, el territorio cubierto por las UBS concentra los establecimientos de prostitución, principalmente en el barrio Jardim Itatinga, que define el paisaje local^(11)^. El barrio se localiza en el sudoeste del municipio, a unos diez kilómetros del centro, en una zona económicamente desvalorizada y que concentra el mayor número de residentes de bajos ingresos^(11)^. Actualmente es considerada la mayor zona de prostitución al aire libre de América Latina, con una circulación aproximada de 2.000 trabajadoras sexuales por mes, lo que permite el funcionamiento de numerosas casas de prostitución de diversos tamaños^(12)^.

Por lo tanto, el área abarcada por la UBS tiene características de exclusión, marginalización y estigmatización de la población y de las trabajadoras sexuales en relación al resto de la ciudad, ya que fue elegida por ser, en la época, un lugar aislado, poco urbanizado y de difícil acceso, marcando este público por la vulnerabilidad social y moral^(12)^. Actualmente, la UBS está situada en la periferia de la zona del territorio que concentra los establecimientos de prostitución, y cuenta con equipos de la Estrategia de Salud de la Familia para ofrecer atención con profesionales del equipo multiprofesional, servicios asistenciales, pruebas de apoyo diagnóstico, vigilancia de la salud y dispensación de medicamentos^(13)^.

Cabe destacar que las investigadoras involucradas en este estudio realizan sus prácticas de investigación, docencia y cuidados de enfermería en diversos establecimientos de salud del municipio, desde la APS hasta los servicios especializados. De esta forma, la atención indirecta y directa a las trabajadoras sexuales ocurre cotidianamente en estos diferentes espacios, trayendo proximidad a la cuestión abordada.

### Población

Participaron en este estudio todos los profesionales del equipo de enfermería de una UBS, incluyendo enfermeros, técnicos y auxiliares de enfermería. El acceso a los participantes se inició con la presencia de la investigadora principal en una reunión de equipo en la UBS, donde se presentó el estudio y se invitó a participar. En esta ocasión, un profesional se inscribió, caracterizándose como el primer participante en el estudio, quien remitió al siguiente profesional que, a su vez, también aceptó participar y remitió al siguiente. Este proceso se repitió hasta que todos los miembros del equipo de enfermería participaron en la investigación.

### Criterios de Selección

Se incluyó a los miembros del equipo de enfermería que tenían experiencia en la atención a profesionales del sexo en la zona estudiada. Se excluyó a los profesionales de baja y de vacaciones.

### Recogida de Datos

Los datos se recogieron en abril y mayo de 2021, utilizando la entrevista fenomenológica, que permite al sujeto que experimenta el fenómeno compartir con el entrevistador el significado de la acción realizada en su mundo de relaciones intersubjetivas^(14)^. Las preguntas disparadoras fueron «¿Alguna vez atendió a una trabajadora sexual en este servicio?», «Cuénteme cómo fue» y «¿Cuál fue su intención al realizar esta atención?», y a partir de ellas se desarrollaron otras preguntas que permitieron al entrevistado profundizar en el tema.

Las entrevistas tuvieron lugar en un lugar privado de las instalaciones de la UBS, realizadas por la investigadora principal, grabadas en audio digital, con una duración media de 15 minutos, sin negativa alguna por parte de los participantes. La recogida de datos finalizó cuando la investigadora se dio cuenta de que el fenómeno había sido desvelado y sus preocupaciones resueltas, y no se habían identificado nuevos elementos en la constitución de las categorías que representan el tipo de experiencia vivida^(15)^. El equipo de investigación se incorporó al BHU durante la recogida de datos y, posteriormente, cuando dio su opinión al servicio sobre el estudio realizado.

### Análisis y Tratamiento de los Datos

Basándose en el marco teórico de la fenomenología social^(9)^, los datos fueron analizados y categorizados por los investigadores que contribuyeron al estudio, sin utilizar*software*. Se analizaron todas las entrevistas. Se siguieron los pasos indicados en los estudios basados en los conceptos de Alfred Schutz^(9)^: transcripción completa de las declaraciones de los participantes; lectura atenta y relectura de las entrevistas para comprender el significado de la experiencia de los profesionales del equipo de enfermería; identificación de los fragmentos que incluyen significados comunes de las experiencias de los participantes; y organización de los discursos en categorías temáticas que demuestren las «razones por las que» y las «razones para» las experiencias de los entrevistados^(9)^. Se recurrió a la fenomenología social y a la literatura científica sobre el tema para llevar a cabo un análisis y una discusión exhaustivos de los resultados presentados en las categorías.

### Aspectos Éticos

La investigación cumplió todos los criterios de la Resolución 466/12, que reglamenta la investigación con seres humanos, y fue aprobada por el Comité de Ética en Investigación de la Universidad Estadual de Campinas, bajo el Dictamen n^º^ 4.659.425. Los participantes en la investigación firmaron el Formulario de Consentimiento Libre e Informado y el Formulario de Autorización de Grabación de Voz antes del inicio de las entrevistas. Para garantizar el anonimato de los participantes, las declaraciones se identificaron con la letra «E» para representar la palabra «entrevistado», seguida del número correspondiente al orden cronológico en que tuvieron lugar las entrevistas.

## RESULTADOS

El estudio contó con 12 participantes, tres enfermeros y nueve técnicos de enfermería, con una media de dos años de trabajo en la unidad para los enfermeros y siete años para los técnicos de enfermería. Las entrevistas permitieron construir la relación «nosotros», organizando las experiencias del otro en dos categorías: la primera destacó los «por qué» del mundo de vida de los participantes, denominada «La percepción del equipo de enfermería sobre el territorio y el servicio de salud»; y los motivos de acción de los participantes se describen en la categoría «La intencionalidad de los cuidados prestados».

### Percepción del Territorio y de los Servicios Sanitarios por Parte del Equipo de Enfermería

Durante las entrevistas, se relataron características del territorio, que corresponde a una zona de prostitución y tráfico de personas, lo que provoca un distanciamiento del profesional en el abordaje e implicación. El territorio determina problemas de salud derivados del consumo y comercialización de sustancias psicoactivas y alcohol. También se reconoce que la población de esta zona está marcada por la vulnerabilidad y la dificultad para mantener los vínculos.

*Aquí no sólo hay prostitución, también hay tráfico de drogas. Así que hay cosas en las que no te puedes involucrar, porque entonces arriesgas tu vida... estamos en un territorio en el que tenemos que tener mucho cuidado con lo que decimos, con lo que hacemos, con cómo nos acercamos a la gente*. (E3)

*Y sabemos que a veces la gente llega sin un problema específico, o no consumen drogas, no son consumidores de drogas o alcohol, y para poder seguir con sus vidas se involucran. Se involucran a un nivel que entra en el tráfico de drogas, que va mucho* más *allá de lo que parece.*(E10)

*Creo que la rutina es la misma que en cualquier otro centro de salud, salvo que la única diferencia es que nuestra población está más expuesta y es más vulnerable*. (E12)

El equipo de enfermería reconoció que su trabajo con las trabajadoras del sexo era mayor cuando el UBS estaba situado en una zona de prostitución. La cercanía del servicio a la población favorecía una atención más cotidiana, pero ahora no hay una atención sanitaria preventiva, y se busca atención cuando hay una queja.

*Antes, cuando estábamos dentro del*[barrio]*, teníamos mucha* más presencia, porque *venían, tenían demanda, estábamos dentro de la prostitución, estábamos dentro de la zona, y hoy no.* (E4)

*Antes el acceso a la atención diaria era mayor. Ahora no, ahora sólo vienen cuando realmente tienen una dolencia. No hay atención preventiva, así que creo que nuestra salida del territorio ha marcado la diferencia. Aunque sigamos allí, pero para ellos, físicamente, ha cambiado.»* (E7)

En cuanto a las características del servicio, se observó que la atención se presta sin necesidad de presentar un documento de identificación, lo que se justifica por el valor que se da a la salud de la persona. El servicio se estructura sobre la base de la demanda y la acogida espontáneas, tratando de atender el mayor número posible de demandas para favorecer la vinculación y el retorno ambulatorio.

*Vienen sin su identidad para ser atendidos... hay varias cuestiones que, si no estamos atentos, intentamos trabajar incluso sin la identidad, porque yo estoy atendiendo a la persona, entonces... sé, si la veo, sé quién es, pero también puede ser que haya mentido sobre su nombre. Así que también tenemos mucho cuidado con este tema del cuidado. Aunque no tenga su identidad, la atendemos y le damos asistencia sanitaria.»* (E3)

*Estas mujeres no suelen venir a recibir atención por programa, siempre vienen por demanda espontánea en la recepción. En general, es una población que no vale la pena reservar. La mayoría de los profesionales son de otros municipios, por lo que nos hacen el seguimiento aquí. Así que intentamos atender en la medida de lo posible la demanda que traen en el día, para intentar vincularlas y traerlas al ambulatorio.* (E1).

El equipo de enfermería señala que las trabajadoras sexuales tienen condiciones de salud frágiles debido a la exposición a ITS, problemas de salud mental, antecedentes de abuso y consumo de sustancias psicoactivas. También menciona que las trabajadoras sexuales no buscan el servicio porque piensan que serán maltratadas, y cuando lo hacen, abandonan el tratamiento o no esperan el resultado de la atención ofrecida.

*Su salud es frágil, ya sabes, por la exposición a las ITS, su salud mental es frágil, suelen ser personas que han sufrido abusos. Hay muchos antecedentes de abusos en este entorno, porque, como he dicho, es muy complicado mantener esta vida nocturna. Así que se encuentran muy perdidos, y el camino es volver a las drogas o al alcohol.* (E10)

*Muchas de ellas ni siquiera vienen porque piensan que van a ser maltratadas... ellas mismos crean esa coraza protectora. Muchas de ellas acaban abandonando el tratamiento. Nosotros vamos a buscarlas*» (E11).

*Como tienen tanta prisa, a veces mientras estamos haciendo el test* rápido, *cuando llamamos para dar el resultado, ya se han ido.»* (E1)

En cuanto a los aspectos culturales, el equipo de enfermería identificó que las trabajadoras del sexo son marginadas y juzgadas por sus familias. También identificaron un bagaje de prejuicios provenientes del núcleo familiar. Señalan que los profesionales de la salud tienen estigmas que hacen que se nieguen a atender a determinados grupos.

*Están muy marginados, se les juzga demasiado, incluso por sus propias familias» (E10).*

*Independientemente de si eres trabajadora sexual o no, venimos con mucho bagaje de nuestra familia; a veces hay prejuicios. (E3)*

*Tenemos este estigma de separar a las personas por su aspecto, por lo que son, porque he trabajado en varias unidades y hay mucho de «Yo no trato a pacientes de salud mental, yo no trato a homosexuales». Porque no son sólo los auxiliares de enfermería y los técnicos los que necesitan formación, porque he visto a profesionales de la educación superior, médicos, hacer esta distinción. (E6)*

En cuanto a las características del trabajo de las profesionales del sexo identificadas por el equipo de enfermería, destacan ir al trabajo acompañadas de un «esbirro», limitar el tiempo que pasan allí y mantener su identidad en la casa donde trabajan. También señalan condiciones de trabajo caracterizadas por el encierro y el pago de multas por ausencias.

*Suelen acudir con el matón de la casa. Así que tienen un tiempo determinado para ser atendidas. Así que si llego tarde, es urgente, no esperan, se van. Hay algunas chicas cuya identidad se guarda en la casa»* (E3).

*Están sometidas a las* más diversas condiciones de *trabajo; a veces están encarceladas. Incluso porque el tiempo que están fuera, tienen que pagar una multa por no estar en la casa o viene alguien a vigilarlas*». (E1)

En cuanto a las demandas de las trabajadoras sexuales, se observó que las quejas están relacionadas con problemas ginecológicos que podrían impedirles seguir trabajando, condiciones infecciosas, búsqueda de medicamentos, violencia, accidentes con preservativos, búsqueda de pruebas rápidas y embarazos no deseados.

*Vienen a nosotros por problemas ginecológicos, violencia o accidentes con el preservativo.* (E12)

*Las prostitutas nos llegaban muy malheridas; a veces las tiraban de un coche, ya sabes... llegaban todas despellejadas y nos ocupábamos de ellas.* (E8)

*Esta es la entrada, porque la exposición era, voy a tomar la medicación, pero la puerta principal es la prueba* rápida. (E10)

*Siempre vienen. A veces vienen con un embarazo que no quieren.* (E2)

*A veces tiene dos días, huele mal, tienes que tomar antibióticos porque ya te duele, ya tienes el proceso infeccioso ahí* [...] *y otra cosa es que, durante la regla, usan algodón dentro de la vagina para poder tener relaciones sexuales. Así que es habitual que venga una chica que no ha conseguido quitarse el algodón, para que se lo saquemos»* (E3).

El equipo de enfermería reconoce que hace su trabajo lo mejor posible, sin discriminación, en cuanto a la acogida, la escucha, la historia clínica personal y familiar, la resolución de las demandas y quejas de las trabajadoras del sexo, la promoción de la salud y el asesoramiento sobre los riesgos. El equipo también adopta un enfoque para comprender la historia del paciente, por qué ha acudido a la unidad y si necesita ayuda psicológica.

*Mi trabajo consiste en acogerlas, escucharlas e intentar resolver sus problemas junto con ellas. [...] tenemos que acogerles, escucharles e intentar promover su salud. Orientarles sobre los riesgos, eso es todo. (E2)En el ámbito de lo que vienen a vernos, las atendemos, tratamos de atenderlas de la mejor manera posible, sin discriminarlos, ¿no?*

*Se ve al paciente, temas de salud, antecedentes familiares, qué ha tenido, qué tipo de enfermedades, se pregunta por las vacunas, se hace de todo, y luego se pasa a la queja principal del paciente.* (E12).

*En primer lugar, nos acercamos a ellos, tratamos de entender un poco su historia, por qué están buscando el servicio. [...] Así que intentamos entender cómo es para ella. Si todo está bien, si necesita ayuda psicológica. (E3)*

Los profesionales llevan a cabo los procedimientos de acuerdo con la queja de la paciente, proporcionan una variedad de pautas y buscan activamente continuar y finalizar el tratamiento.

*Ponemos el espéculo, sacamos el algodón, aconsejamos sobre el riesgo, aconsejamos sobre la copa menstrual. Así que es una amplia gama de consejos ginecológicos»* (E1).

*Problemas con las pruebas, que hacemos para las ITS, nos ocupamos de las ITS, damos instrucciones.*[...] *como he dicho, hay que ir tras ellas, porque la mayoría no vuelven. Entonces nosotros somos los que nos quedamos atrás, tenemos que estar pendientes para que ellos puedan terminar su tratamiento.»* (E11)

### La Intencionalidad de la Atención Prestada

Con relación a la expectativa de los cuidados prestados, el equipo de enfermería espera promover la salud de las trabajadoras sexuales, reducir el riesgo de transmisión de enfermedades, acogerlas, garantizar que vuelvan para el tratamiento y que tengan una calidad de vida satisfactoria. También esperan que los cuidados sean resolutivos y marquen una diferencia en la vida de esa persona. Por último, esperan que mejoren los cuidados y la protección, que disminuya la exposición y que comprendan la necesidad y el derecho a cuidar su cuerpo.

*Espero que promueva el bien, que promueva la salud para ellos.* (E2)

*Reducir el riesgo de transmisión de enfermedades... también se sienten bienvenidas* [...] *y reciben retroalimentación, tienen dónde buscar lo que necesitan*. (E5)

*Éxito.*Para que *puedan tener una mejor calidad de vida.* (E10)

*Espero que sea resolutivo y que marque una diferencia en la vida de esa persona.* (E1)

*Esperamos que puedan cuidarse mejor, protegerse mejor, no exponerse tanto.*[...] *Pero ya está, ella intenta entender que tiene que cuidar su cuerpo. Aunque sea una profesional, tiene derecho a cuidar su cuerpo*» (E12).

## DISCUSIÓN

Los profesionales de enfermería que participaron del estudio relataron sus experiencias en la atención a trabajadoras sexuales en el área de cobertura de la UBS, siendo demarcada la presencia de tráfico de drogas y prostitución en la zona, lo que influye en la actitud del equipo de enfermería en la atención a esta población. Toda acción trae una expectativa de respuesta, y en este escenario, es posible ver el territorio como el espacio de encuentro subjetivo del mundo de vida del profesional y de las personas que existen en él, como las trabajadoras sexuales^(9)^.

El territorio es dinámico y, a partir de las relaciones creadas, puede ser producto o productor de acciones. Por lo tanto, al leer la realidad que se le presenta al individuo, se lo invita a presentar la forma en que se ubica en el territorio, conocida como la actitud natural^(9)^. A través de ella, es posible abordar el cuidado en lugares que pueden causar tensión y miedo a los profesionales de salud, debido a la presencia de violencia^(16)^.

El territorio en APS se define como un espacio geográfico delimitado con una población inscrita, dinámica y en constante cambio, sujeta a múltiples variables, como las vulnerabilidades sociales^(16)^. En este espacio, el proceso salud-enfermedad se produce como resultado de los determinantes sociales de la salud, caracterizados como factores relacionados con las condiciones de vida y de trabajo de un individuo, que pueden influir en la aparición de problemas de salud^(16,17)^. De este modo, el territorio de las UBS en este estudio es el resultado de una dinámica histórica en constante movimiento que requiere su reconocimiento para la prestación de asistencia sanitaria, ya que sus orígenes se remontan a la segregación social y a las estrategias higiénicas de los grandes centros urbanos.

Sin embargo, los profesionales de salud de la UBS relataron dificultades para trabajar en este escenario, como barreras para acceder a la población, permanecer presentes en el territorio y mantener los vínculos. El territorio cubierto por la APS puede encontrarse con otras divisiones diferentes de las organizaciones populares, como el narcotráfico, pero es importante considerar que la población que vive allí tiene necesidades de salud y cuidado, lo que requiere el reconocimiento y la planificación de las acciones a desarrollar^(16)^.

Los participantes mencionaron que antiguamente la UBS estaba ubicada entre casas de prostitución, lo que representaba una estrategia de acceso y proximidad de la población a los servicios de salud. Sin embargo, en la organización actual, la atención sólo se realiza a través de denuncias, ya que requiere que las trabajadoras sexuales se desplacen hasta el servicio de salud. Se recomienda que la ubicación de las UBS tenga en cuenta aspectos geográficos para facilitar el acceso de la población, lo que muchas veces no es posible, y las unidades acaban ubicándose en zonas de difícil acceso^(18)^.

Por lo tanto, corresponde a los profesionales de enfermería colaborar en la construcción de estrategias para reducir las barreras de acceso de la población vulnerable, empezando por reconocer el territorio cubierto por el servicio de salud e identificar a las personas que viven en él. Los participantes en el estudio mencionaron las acciones de acogida basadas en la demanda espontánea y la no imposición de la presentación de documentos de identificación como formas de acoger y garantizar el acceso de las profesionales del sexo a las UBS. Cabe destacar que asegurar esas estrategias es esencial para garantizar el derecho de las personas al SUS, como resultado de las inversiones en el proceso de trabajo de equipos multiprofesionales, cuya presencia activa es indispensable, ya que juntos buscan establecer una relación de confianza con los usuarios, promoviendo la construcción de soluciones y formas de atención^(19)^.

Además, los participantes señalaron que las necesidades de salud de las trabajadoras sexuales están permeadas por la exposición a las ITS, el abuso de sustancias psicoactivas, la violencia y los problemas de salud mental. Los estudios demuestran que los problemas de salud mental de las trabajadoras del sexo suelen estar mediados por el lugar donde trabajan y su vulnerabilidad a la violencia, el abuso de sustancias y el estigma^(1,20)^. Las ITS son un factor agravante para la salud mental de esta población, ya que son un fenómeno complejo y multifactorial cuyas principales medidas están dirigidas a la prevención, detección temprana y tratamiento oportuno, potenciadas por el acceso a los servicios de salud^(21,22)^. Todos estos eventos asociados y vividos por las trabajadoras sexuales conforman su biografía de vida, aspecto importante para el equipo de enfermería al momento de pensar en estrategias para garantizar el acceso a la atención de esta población, considerándolos como determinantes sociales que influyen directamente en su salud.

Sin embargo, las entrevistadas también señalaron que las trabajadoras sexuales muchas veces no acuden a los servicios de salud porque piensan que serán maltratadas o irrespetadas, cuyo resultado directo es el abandono del tratamiento o la interrupción de la atención. En su trabajo, las trabajadoras sexuales son entendidas en términos de vulnerabilidad, y es posible ver que los principales marcadores de vulnerabilidad social en esta población están relacionados con los datos socioeconómicos, el cotidiano de la profesión, el prejuicio social, el miedo a buscar servicios de salud debido al estigma y a los profesionales de salud no calificados para prestar atención^(1,23)^.

Se puede entender que ese comportamiento es el resultado de los aspectos biográficos de lo que esas personas han vivido, cuyas experiencias previas de violencia y exposición han constituido su cuerpo de conocimientos^(9)^. Como ejemplo, el equipo de enfermería participante cita que algunos aspectos culturales conducen a la marginación y al juicio social, incluso por parte de sus propios familiares. El trabajo sexual ha estado históricamente vinculado al juicio moral de la sociedad, que comienza con los prejuicios de la familia y se extiende a los profesionales sanitarios^(24,25)^. El apoyo y el rechazo de la familia pueden enumerarse como factores agravantes o protectores, en los que los resultados negativos para la salud, influidos por el rechazo y la falta de apoyo, conducen a intentos de suicidio, depresión y consumo de sustancias^(24,25)^. Sin embargo, pocos profesionales del sexo mantienen vínculos sociales con sus familias, lo que intensifica su vulnerabilidad. Sin embargo, el apoyo social y los servicios sanitarios pueden maximizar las intervenciones que ayudan a reducir la vulnerabilidad^(24,25)^. En este contexto, es esencial que las enfermeras proporcionen cuidados libres de creencias personales, valores morales o juicios, priorizando la construcción de relaciones de confianza y el fortalecimiento del apoyo social e institucional, elementos esenciales para reducir la vulnerabilidad, fomentar la adherencia a los cuidados, minimizar los impactos negativos sobre la salud y garantizar una atención integral.

Los participantes señalaron aspectos que caracterizan parte del mundo de la vida y la biografía de las trabajadoras sexuales, como ir a la UBS acompañadas de un secuaz, que les retengan sus documentos personales o que las encarcelen en el lugar de trabajo. Las condiciones denunciadas impregnan el proceso salud-enfermedad de las trabajadoras sexuales, representando una rutina diaria de ausencia y violación de necesidades humanas básicas y derechos mínimos, como el acceso a la salud^(1,3)^.

En el umbral entre la criminalización y la regulación del trabajo sexual, experiencias como la de la Unión Europea indican que la criminalización aumenta la marginación y la estigmatización, y esto conlleva riesgos aún mayores de problemas de salud, como la exposición a ITS^(26)^. Sin embargo, son necesarios cambios estructurales en la legislación que garantice los derechos laborales y políticas que aborden los derechos humanos, combatiendo el estigma y la discriminación^(26)^. Con base en esto, se enfatiza que las enfermeras pueden actuar en estos escenarios para promover el cuidado respetuoso y la mediación en el acceso a la salud de las trabajadoras sexuales, identificando situaciones de riesgo y vulneración, garantizando los derechos básicos y sensibilizando a los gestores sobre políticas inclusivas que combatan el estigma, la vulnerabilidad y promuevan la salud integral.

Como resultado de la dinámica de trabajo que viven las trabajadoras sexuales, están las demandas que ellas traen cuando llegan a la UBS y son recibidas por el equipo de enfermería: desde quejas de condiciones ginecológicas que ya suponen un riesgo para la salud hasta la búsqueda de apoyo para situaciones de violencia. Así, los miembros del equipo de enfermería destacaron en el estudio que acogen, escuchan, orientan y promueven la salud de las trabajadoras sexuales que llegan a la unidad. Destacamos que esas acciones de cuidado utilizadas por el equipo de enfermería en el acceso a las trabajadoras sexuales se organizan a través de la relación cara a cara, pues es en el encuentro con el otro que se identifican las necesidades de cuidado, con la posibilidad de iniciar la construcción de un vínculo^(9)^.

Aún con relación al papel de la enfermería en las UBS, el trabajo se extiende más allá del primer contacto con el paciente, en el cual se recomienda que sus demandas sean evaluadas a partir de una perspectiva interprofesional y en espacios colectivos que potencien la acción conjunta en la planificación de los cuidados^(27)^. Además, con el desafío del seguimiento y retorno de las trabajadoras sexuales a la UHB, es necesario buscarlas activamente para reforzar su vinculación al servicio, evaluar su estado general de salud y orientarlas en la prevención de diversos problemas de salud^(27)^. Por lo tanto, se recomienda que el equipo de enfermería participe de espacios de discusión de casos y lidere acciones que apoyen el vínculo entre la UBS y las trabajadoras sexuales para identificar sus necesidades de salud y ampliar el alcance de la atención a través de la búsqueda activa, visitas domiciliarias y acciones de promoción y prevención de la salud en el área.

Es posible que el uso de la búsqueda activa como estrategia de retorno a la UBS explique la marginación y juzgamiento de las trabajadoras sexuales. La falta de reglamentación de la práctica profesional refuerza el mantenimiento de la vulnerabilidad, la invisibilidad en las políticas públicas gubernamentales, la ausencia de protagonismo social, los estigmas y la ocurrencia de la explotación sexual^(1,5)^.

Cabe destacar que los elementos de tensión originados en el mundo del trabajo son importantes para la comprensión del mundo de vida de esta población, y exigen una actitud natural, que promueva la resistencia a los cuidados interpretados por el equipo de enfermería a través de una lente abolicionista, realidad que necesita ser transformada invirtiendo en relaciones cara a cara, que puedan favorecer el conocimiento del mundo de vida de estos sujetos y la apertura al cambio^(9)^. A veces, los cuidados prestados por el equipo de enfermería se dirigen a las partes del cuerpo que corresponden a la naturaleza del trabajo de las trabajadoras sexuales, y esta población es entendida desde el punto de vista del establecimiento de relaciones sexuales, lo que limita la atención a las cuestiones sexuales y reproductivas, la prevención de las ITS y la anticoncepción^(5)^.

Sin embargo, el equipo también desarrolla acciones que contribuyen a la subjetivización de estas profesionales, colocándolas como protagonistas en la producción de salud. Esto ocurre cuando basan la atención en la relación cara a cara, basada en la escucha activa y en el conocimiento de los antecedentes familiares, comprendiendo la historia y ofreciendo ayuda psicológica^(9)^. Así, es posible indicar que existe un conflicto en relación a la actitud natural del cuerpo como objeto de intervención en el cuidado de enfermería, al entender a las trabajadoras sexuales solamente desde el punto de vista de su actividad laboral, porque, al mismo tiempo, hay importantes acciones de cuidado que avanzan en relación a esta comprensión destacada, lo que permite apuntar a las tecnologías blandas como estrategias de subjetivación con potencial para transformar el mundo de vida de las trabajadoras sexuales a través del cuidado de enfermería.

En la relación cara a cara, el equipo de enfermería muestra intenciones en el cuidado de las trabajadoras sexuales, promoviendo la salud, construyendo vínculos para garantizar que ellas vuelvan al servicio, mejorando su calidad de vida y concientizando sobre los derechos que tienen sobre sus cuerpos. Este abordaje demuestra motivación para ofrecer cuidados resolutivos y transformadores, en consonancia con las necesidades de salud de esta población y con el objetivo de la APS de promover la atención integral en el territorio^(8,18)^.

El deseo de bienestar en relación con los demás forma parte de la actitud natural del proceso de trabajo de enfermería, cuya finalidad es desarrollar la acción de cuidar a los demás, y la salud va más allá de estar enfermo o no, presentándose como una consideración plural del individuo que se establece en el mundo de forma biopsicosocial^(8)^. Por lo tanto, el equipo de enfermería, a partir de su cuerpo de conocimientos construidos a lo largo de su formación profesional, reconoce el derecho de las trabajadoras sexuales al cuidado basado en políticas públicas. Al alinear sus intenciones con las necesidades de salud de esta población, demuestran motivación para desarrollar acciones de enfermería que resuelvan y transformen esta perspectiva, reafirmando el papel de la APS en la promoción de un cuidado integral, humanizado y centrado en la perspectiva biopsicosocial.

Este estudio destaca las acciones y contribuciones de la enfermería en la APS, enfatizando su papel esencial en la atención a las trabajadoras sexuales, incluso frente a los desafíos impuestos por la marginalización social de esta población. Destaca la importancia de la actuación interdisciplinar y multiprofesional, construyendo lazos y promoviendo la autonomía, incluso en condiciones de trabajo adversas y frente a barreras burocráticas. Además, la enfermería puede desempeñar un papel en la implementación de políticas públicas inclusivas, proponiendo estrategias que aumenten el acceso y garanticen la continuidad de los cuidados, reforzando los principios de la APS de integralidad y centrado en la persona.

Con relación a las limitaciones del estudio, señalamos que se trata de un municipio específico y que la complejidad del tema estudiado involucra otros determinantes sociales para la lectura del mundo de la vida, que este estudio no se propuso analizar. También es necesario ampliar las investigaciones sobre el tema, considerando la intersección con otros marcadores sociales de diferencia y la adopción de abordajes metodológicos variados. Esto se debe a que la perspectiva fenomenológica, al favorecer un análisis profundo y detallado de los matices de la experiencia vivida por los participantes, se caracteriza por priorizar la calidad y la riqueza descriptiva de los datos sobre el tamaño de la muestra^(15)^. Este enfoque subraya que el rigor y la profundidad en la comprensión del fenómeno estudiado están intrínsecamente ligados a la capacidad de captar y describir detalladamente las experiencias de los participantes^(15)^.

## CONCLUSIÓN

En este estudio se constató que la percepción del equipo de enfermería sobre los cuidados prestados a las profesionales del sexo considera sus acciones a partir de aspectos de su biografía vital y del conjunto de conocimientos sobre sus experiencias vividas.

Se constató que el trabajo del equipo de enfermería en APS está permeado por la dinámica del territorio en que actúa, marcado por la presencia del narcotráfico y de la prostitución, imponiendo desafíos adicionales a la práctica del cuidado. El reconocimiento de las vulnerabilidades enfrentadas por las trabajadoras sexuales resalta la necesidad de acciones interdisciplinarias y estrategias que promuevan el acceso y la continuidad de la atención, incluso frente a las barreras impuestas por la marginalización social.

Los hallazgos del estudio también revelan que el equipo de enfermería desempeña un papel fundamental en la construcción de vínculos, utilizando tecnologías de atención que buscan superar las barreras burocráticas.

Sin embargo, la precariedad de las condiciones de trabajo, la falta de regulación de la práctica profesional y la invisibilidad social dificultan la búsqueda de atención sanitaria por parte de las trabajadoras sexuales. Se recomienda que los servicios de salud construyan estrategias para facilitar el acceso y el derecho a la salud, así como políticas públicas más inclusivas.

Por último, la relación cara a cara establecida entre el personal de enfermería y las trabajadoras sexuales revela una ambigüedad entre la actitud natural que limita la atención a las cuestiones sexuales y reproductivas y la promoción de la subjetivación, que reconoce a estas personas como protagonistas de su salud. La motivación del equipo de enfermería para promover la autonomía y el bienestar en la vida del otro refleja la esencia de la APS como puerta de entrada al SUS, en la cual la atención debe ser resolutiva, integral y centrada en la persona.
